# Utilizing additive manufacturing and mass customization under capacity constraints

**DOI:** 10.1007/s10845-022-02007-x

**Published:** 2022-09-27

**Authors:** Rachel Lacroix, Anna Timonina-Farkas, Ralf W. Seifert

**Affiliations:** 1 École Polytechnique Fédérale de Lausanne (EPFL), Technology and Operations Management Chair, Odyssea Station 5, 1015 Lausanne, Switzerland; 2 IMD Business School, Chemin de Bellerive 23, P.O. Box 915, 1001 Lausanne, Switzerland

**Keywords:** Manufacturing, Mass Customization, Inventory Policy, Pricing, Product Life Cycle

## Abstract

Additive manufacturing (AM), originally used for prototyping, is increasingly adopted for custom final part production across different industries. However, printing speed and production volume are two barriers for the adoption of AM for product customization at large scale. Nevertheless, manufacturers could aim to combine the benefits of AM for product customization with traditional mass customization (MC) technologies over the product life cycle (PLC). This approach is showcased in our paper as a manufacturing opportunity and is addressed via a non convex-concave optimization model that considers a monopolist manufacturer producing horizontally differentiated products at scale. To satisfy individual customer preferences under capacity considerations, the firm jointly decides on the inventory, production quantity, product variety, optimal technology-switching times (between AM and MC) and pricing strategy. Our approach can be implemented by decision-makers to leverage customer-centricity and benefit from this novel hybrid manufacturing practice. By deriving a closed-form solution for the production quantity based on an adaptive inventory policy, the resulting optimization problem is solved using the Sample Average Approximation framework grounded by analytical results. Our results demonstrate that the new usage of AM with MC can benefit a manufacturer for customer-centric driven strategies. Significant profit improvements can be achieved with an AM–MC–AM technology-switching scenario under certain capacity conditions and with an increasing-decreasing pricing strategy. Our results also indicate that the benefits of pricing flexibility are highest when capacity is unlimited or when the firm does not hold inventory. Under capacity constraints, a simple decreasing pricing policy combined with inventory performs very well.

## Introduction

Technological advances and digital transformation influence and shift common practices in different fields, including the field of *additive manufacturing* (AM, also known as 3D-printing). Indeed, AM is being used for product prototyping already since 1988 (Hon, 2007). Nevertheless, only recently has it been adopted for rapid manufacturing (RM) in the serial production of final parts (see Campbell et al. Campbell et al. 2020). Adopting AM for final parts production has been proliferating across different industries (Berman, 2012): In the automotive industry, BMW is manufacturing 3D-printed customized components for commercial vehicles. AFMG (2020) reports that “from consumer electronics to toys and sportswear, key players within the consumer goods industry are increasingly recognizing 3D-printing as a valuable addition to existing manufacturing solutions.” As for industrial goods, Bowman International, a leading UK bearings manufacturer, and MX3D, a Dutch company that 3D-prints aluminium bike frames, are a few other examples of 3D-printing for large-scale end-part production (Davies, 2018; MX3D, 2020). Overall, according to the industrial report by Campbell et al. (2020), rapid manufacturing using AM grew from 3.9 to 60.6% of the total AM market during recent years, while more and more manufacturers are interested in using 3D-printing technologies for full-scale production as they believe they can benefit from product *customization* at lower costs. In this type of AM manufacturing, customization implies a very high flexibility of manufacturing systems and, thus, the ability to address customer needs delivering high speed and lower cost which are both at the forefront of AM technology. Following this path in our article, AM is referred to as the technology fully adoptable for rapid production of perfectly-customized final goods (Deradjat and Minshall, 2017). The absence of tooling requirements, geometry freedom, and inventory reduction through just-in-time operations makes AM particularly attractive over conventional manufacturing processes (Weller et al., 2015; Baumers et al., 2016).

Unlike AM, the technology of *mass* “customization” (MC) has the unique ability to design and manufacture products at mass production efficiency and speed (see Anderson, 2004). In this paper, MC is considered as the technology which aims to address as large a customer base as possible via a limited assortment of products and with a lower setup cost than AM Alptekinoğlu and Corbett (2008), Berman (2012) and Dong et al. (2020). Although AM and MC are capable of producing custom final parts cost-effectively, these two processes display technology-specific cost structures and different customization capabilities highlighted in Lacroix et al. (2021). According to them, the technologies differ in (i) the part production process, (ii) the degree of part customization, (iii) the setup cost and (iv) the marginal cost. This is based on the fact that AM is commonly used to manufacture complex geometrical parts in one production run, unlike MC, which requires several production tools for an equivalent result. By this, AM technology offers an unlimited assortment of product variants (and, thus, allows for perfect product customization), while MC displays a restricted variant assortment only (Dong et al., 2020). Furthermore, similar to the work of Weller et al. (2015), one can assume a constant marginal cost under AM as opposed to a linearly increasing cost in the number of product variants offered under MC.

Currently, AM technology is not widely deployed for large-scale production and is not expected to replace traditional MC processes. In particular, printing speed and production volume are preventing AM adoption on a large scale (Arbabian and Wagner, 2020). Nevertheless, researchers and industry experts argue that AM can supplement existing MC processes for the benefit of manufacturers (Holweg, 2015; Rogers et al., 2016; Sasson and Johnson, 2016; AFMG, 2020). In this article, the benefits of combining AM with traditional MC processes over the course of the product life cycle (PLC) are analyzed. For this, both the demand and the supply sides are considered in order to provide quantitative decision tools to assess the optimal use of AM and MC technologies.

**Fig. 1:**
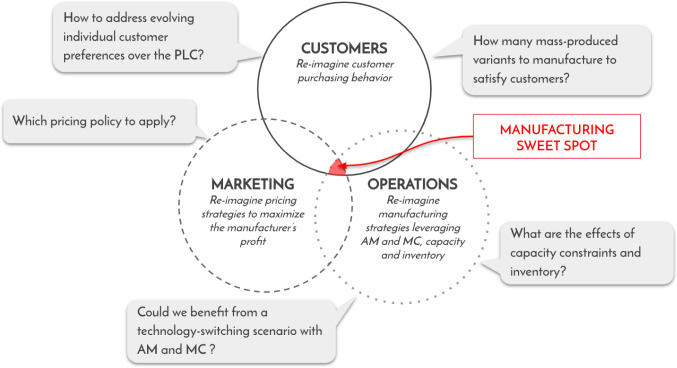
Manufacturing sweet spot to transition toward mass customization at scale

On the demand side, practitioners and academics have scrutinized customer-centric strategies, recognized to add business value, particularly in the context of MC. For instance, (Lacroix, 2021) develop a time-varying locational customer choice model that allows for customer heterogeneity and forward-looking behavior. They highlight the importance of linking individual customers’ preferences with the PLC and the technology choice (AM or MC) over time. Following their path, the combination of customer-centric strategies with the use of AM and MC provide broad manufacturing opportunities. Yet, economic benefits of particular strategies still need to be uncovered. This especially holds true for the cases with capacity constraints and across the PLC. As demonstrated by Dong et al. (2020), AM and MC technologies present different degrees of flexibility and cost structures. Hence, optimal technology-switching scenarios operating AM and MC over the PLC and with limited capacity are of interest to manufacturers who aim to maximize their profit while addressing individual customer preferences. The well-known design thinking Venn diagram (Ideo, 2020) is widely used in practice to deliver a profitable customer-centric solution. Building on it (see Fig. 1), one can illustrate the manufacturing *sweet spot* that drives operational efficiency, customer satisfaction, and profit.

The diagram also highlights pending questions related to this opportunity: Which pricing policy should be applied? How many product variants to manufacture to satisfy individual customer preferences? Which technology-switching scenario over the PLC (i.e., combining AM with the firm’s existing MC processes) would be most beneficial? What are the effects of production capacity constraints and inventory decisions on marketing and operations decisions? This paper aims to answer the following question: *“How can a manufacturer best combine the benefits of AM with traditional MC technology under capacity constraints?”*. Using a mathematical model, which accounts for both the individual customer preferences and the manufacturer’s capacity constraints throughout the PLC, the impact of combining AM with MC for a monopolist manufacturer producing horizontally differentiated products at scale is analyzed. In this model, the manufacturer jointly decides on the inventory, production quantity, product variety, technology-switching times (between AM and MC) and product prices. To this end, several technology-switching scenarios, and three production capacity and inventory cases are analyzed.

On the supply side, the manufacturing setup presented in this paper demonstrates that significant profit improvements can be achieved with an AM–MC–AM technology-switching scenario given our manufacturing setup, revealing that to consider both customer heterogeneity and limited production capacity requires an increasing-decreasing pricing policy. The findings naturally confirm that the benefits of pricing flexibility are the highest when capacity is unlimited or when the firm does not hold inventory. Nevertheless, under capacity constraints, a simple decreasing pricing policy combined with inventory performs very well and lessens the need for pricing flexibility. Overall, our numerical results show that combining AM and MC with customer-centric marketing and operations strategies can increase a manufacturer’s profit while addressing individual customer preferences.

The remainder of this paper is organized as follows. “Literature review” section explores the relevant literature and in “Model framework” section our analytical model is described in detail. Specifically, the customer choice model is presented, various manufacturing scenarios are introduced, and the technology production characteristics that differentiate AM from MC are analyzed. Next, our demand forecasting methods are described and our objective function is characterized by building on the analytical properties of our demand forecasting method to ground the non-convex-concave optimization problem. In “Solution approach to maximize profit” section, an adaptive inventory policy is developed and the Sample Average Approximation (SAA) framework is used to solve our optimization problem numerically. “Numerical experiments” section is dedicated to robustness tests and sensitivity analysis for the validity of the SAA framework, presenting the main managerial insights thereafter. “Conclusion and managerial insights” section summarizes our key findings. The e-companion provides an overview of our notations and parametric assumptions (see Table 7), as well as analytical results, algorithms, and proofs.

## Literature review

A growing body of literature develops analytical models to evaluate the impact of AM vs. conventional manufacturing systems on operations management (Westerweel et al., 2018b, a; Sethuraman et al., 2018; Song and Zhang, 2020; Dong et al., 2020; Chen et al., 2020). Early works in this field focused primarily on spare part logistics (Westerweel et al., 2018a; Song and Zhang, 2020), consumer goods retailing (Chen et al., 2020), component design cost analysis (Westerweel et al., 2018b), and assortment planning (Dong et al., 2020). Only few papers (i.e., Dong et al. (2020); Chen et al. (2020)) position themselves at the operations-marketing interface, considering both the demand and supply perspectives. Our work contributes to this literature stream.

In the work of Chen et al. (2020), the authors focused on AM adoption cases in a dual-channel retail setting (i.e., online and in-store channels) and studied the firm’s joint decision about product offers, pricing and inventory. Further, Dong et al. (2020) were among the first to evaluate the impact of AM over conventional manufacturing systems on a firm’s manufacturing strategy. The authors examine three manufacturing technologies (i.e., AM, traditional flexible, and dedicated technologies) and focus on product assortment decisions under capacity constraints, demonstrating that pairing AM with dedicated technology allows wider product variety and profit increase. Recently, Lacroix et al. (2021) has built on the work of Dong et al. (2020) to add technology-switching (between AM and MC) and pricing decisions under PLC considerations to the optimal decision-making process. Assuming limited capacity under AM and MC, our article extends their work to account for inventory decisions under MC technology.

As our research focuses on a monopolist manufacturer producing custom products that are horizontally differentiated, papers modeling a utility-based demand in the mass customization (MC) literature are relevant and of interest to us. Commonly used in the marketing literature, utility-based demand models in assortment planning (see Kök et al., 2015) for a detailed review of demand models in this research area) consider customer heterogeneity. Although some researchers (e.g., Dong et al. (2020)) model customer preferences through a multinomial logit (MNL) model, in our paper demand is derived from the “*Hotelling-Lancaster-Bass*” (HLB) demand model developed by Lacroix et al. (2021). The HLB model is a novel time-varying locational customer choice model that combines the classic Hotelling-Lancaster model (Lancaster, 1990) (also referred to as an “address model” by Kök et al. (2015)) and the well-known Bass diffusion model (Bass, 1969). For tractability reasons, most of the above papers study marketing and operations decisions in a static setting. However, forward-looking customers are typically variant-sensitive but also time-sensitive in their purchasing decisions. Thus, as previously mentioned, our article adopts the HLB demand model, which characterizes the demand of heterogeneous customers at the individual level and also mimics the product life cycle (PLC) dynamics.

With respect to the operations side, few studies have been conducted on generalized Bass diffusion models (i.e., including the selling price) (Bass, 2004) with production or inventory decisions e.g., Ho et al. (2002), Kumar and Swaminathan (2003) and Shen et al. (2013). Ho et al. (2002) jointly analyze demand and sales dynamics in a constrained new product diffusion context where backorders and lost sales are deemed. Kumar and Swaminathan (2003) explicitly model interactions between manufacturing and marketing decisions for a firm with a fixed production capacity. Shen et al. (2013) focus on the joint impact of pricing, sales, and production decisions with limited capacity. They derive optimal policies for handling new product introductions. Unlike these papers, our article does not use the Bass diffusion model as such to model the demand but includes it through the HLB framework. This goes in line with the fact that most Bass diffusion models consider aggregate demands and do not address the needs of operations managers to apply customer-centric operations strategies.

The pioneering work of Chatterjee and Eliashberg (1990) developed an innovation diffusion model using a micro-modeling approach (i.e., modeling demand at the individual level) and highlighted the added-value of customer segmentation in terms of adoption times. Lacroix et al. (2021) showed the importance of modeling time-varying customer preferences at the individual level as they could directly impact the operations, marketing decisions and manufacturers’ profit. Accordingly, since our focus is not on new product introduction timing but on evaluating the benefits of combining AM with traditional MC, our work builds upon that of Lacroix et al. (2021). The authors consider a monopolist manufacturer who jointly optimizes technology-switching, pricing and product variety decisions across the PLC, whereas our model considers AM and MC technologies as capacity-constrained with inventory decisions under MC technology.

Our work is also related to the literature on pricing and production control under capacity constraints. While this line of literature primarily focuses on inventory control where a demand distribution is assumed to be known and stationary, few studies are intended for consumer goods exhibiting non-stationary demand (i.e., the demand probability function changes over time) and partial information. The focus in this paper is on adaptive inventory control problems for non-stationary demand and incomplete information. The earliest model investigating stochastic non-stationary demand was presented by Hadley and Whitin (1961). They proposed an optimal inventory model where demand is Poisson distributed. Graves (1999) developed an adaptive base-stock inventory policy for a non-stationary problem. However, in Graves’ model, the firm has complete information as the demand is fully characterized by an auto-regressive integrated moving average ARIMA(0,1,1) and by the observed demand from previous periods. Kurawarwala and Matsuo (1996) presented a growth model to estimate the parameters of a non-stationary demand process over its entire PLC but do not revise these estimates using new observations. Treharne and Sox (2002) examined a periodic-review inventory model with non-stationary and partially observed demand. The demand state is estimated using the observed sales in each period. The inventory control problem is modeled as a partially observed Markov’s decision process. Recently, Yang and Kim (2018) developed a joint replenishment policy characterized by a variable order-up-to level for items sold in a retail system. They adopt a multiplicative seasonal model to generate demand data to forecast the true demand and assume that the forecast errors are normally distributed. Our model forecasts the demand using a discrete-time version of a diffusion model. The above-mentioned studies are different from our work in that they do not apply customer-centric manufacturing strategies, that is, they do not consider the evolving customer purchasing behavior at the individual level and most of them do not combine marketing and operations decisions. Key realistic features that distinguish our paper from the above-mentioned ones are (i) customer heterogeneity in terms of product attributes and buying times, (ii) pricing flexibility, and (iii) capacity constraints and PLC considerations at the marketing-operations interface. In fact, technology-switching, pricing, product variety, and inventory decisions that are of interest to operations managers are jointly optimized in our work, in a context where customer-centric operations strategies have gained much attention.

## Model framework

Consider a monopolist manufacturer, who serves customers over a finite time horizon with periods $t=1,...,T$. The customer preferences are heterogeneous in product attributes and buying times and are described through a time-varying locational choice model similar to Lacroix et al. (2021). This model is referred to as the Hotelling-Lancaster Bass (HLB) model by the authors (see Section 3.1). Accounting for the individual customer preferences, the manufacturer decides which production technologies (AM, MC or their combination) to employ over the PLC in order to maximize profit. The technology-switching scenario uses a specific combination of AM and MC technologies and requires the computation of optimal *technology-switching times*, defined as a pair $(T_{A\rightarrow M},T_{M\rightarrow A})$ where $T_{A\rightarrow M}$ and $T_{M\rightarrow A}$ represent the time periods when the manufacturer changes the AM technology for the MC setup and vice versa. We set $\mathcal {T} = \{(T_{A\rightarrow M},T_{M\rightarrow A}):0\le T_{A\rightarrow M}< T_{M\rightarrow A}\le T+1\}$ and denote AM and MC production periods by $\mathcal {T}^A = \{t \in \{0,T\}: t\le T_{A\rightarrow M}$ or $t>T_{M\rightarrow A}\}$ and $\mathcal {T}^M = \{t \in \{0,T\}: T_{A\rightarrow M}<t\le T_{M\rightarrow A}\}$ correspondingly. To determine those optimally, an analytical model is proposed that jointly accounts for the following decisions over time $t$: **(i)** the technology-switching times; **(ii)** the pricing strategy $(p_t)_{1\le t\le T}$; **(iii)** the product variety under MC, *n*; **(iv)** the production quantity for each mass-customized variant *j*, $Q_{j,t}$.

Starting with the customer choice model, manufacturing scenarios, production technology assumptions and firms’ optimal operational decisions are discussed next.

### Demand side: customer choice model

Let the potential market size, *N*, represent the initial number of potential customers. Unknown a priori, this number can be estimated qualitatively via market research or via the Delphi method (Snyder and Shen, 2019). A *random customer* $\xi$ is determined by two independent attributes $\tau$ and $\phi$: $\xi =(\tau ,\phi )$, $\mathbb {P}_\xi =\mathbb {P}_\tau \otimes \mathbb {P}_\phi$, where $\tau$ is an *ideal buying time* and $\phi$ is the level of the customer’s heterogeneity describing his/her *product preferences*. To satisfy customers, the manufacturer adopts *horizontal product differentiation*, i.e., the selling price is equal for all product variants.

The virtual product space $\Phi =[0,1]$ is a set of customers’ ideal product variants $\phi$, uniformly distributed as $\mathbb {P}_\phi =\mathcal {U}([0,1])$. The AM technology is assumed to serve customers perfectly in product attributes, while, in contrast, the customers are served with the nearest mass-customized variant under the MC technology. The set of MC-produced variants is denoted by $\mathcal {X}=\{x_1,\ldots ,x_n\}\subset [0,1]^n$ and contains $n$ products. Further, the customer’s ideal buying time $\tau$ follows the *truncated Bass distribution* $F_{\tau }(t)$, which models the spread of customers along with the PLC (see Lacroix et al. 2021). Note that the Bass distribution (Bass, 1969) models the PLC using coefficients *p* and *q* describing innovators and imitators correspondingly. The truncated Bass distribution is the modification allowing to consider a finite time selling horizon as in this paper.

Overall, the customer $\xi$ acquires the following utility at period *t* from a given production technology:

1 $$\begin{aligned} U^{\mathcal {T}}(\xi ,t) =U^{\mathcal {T}}(\tau ,\phi ,t)= & {} \omega (\tau )\left( 1-\gamma (\tau )\frac{\vert \tau -(2t-1)/2\vert }{T}\right. \nonumber \\&\quad \left. -\lambda (\tau )d(\phi , \mathcal {X})\mathbf {1}_{\mathcal {T}^M}(t)\right) . \end{aligned}$$

Here, $\omega (\tau )$, $\gamma (\tau )$ and $\lambda (\tau )$ are the customer’s *willingness-to-pay*, *time-* and *product-sensitivities*, correspondingly. Further, ${d}(\phi ,\mathcal {X})$ is the Euclidean distance between the customer’s ideal variant $\phi$ and its nearest mass-customized variant in $\mathcal {X}$, where the set $\mathcal {X}$ is a finite subset of $[0,1]^{n}$ and $\phi$ is an element uniformly drawn from the interval [0, 1]; $\mathbf {1}_{\mathcal {T}^M}(t)$ denotes the indicator function equal to one under the MC technology and to zero, otherwise.

The *product disutility*, which is equal to the product sensitivity $\lambda (\tau )$ multiplied by the product misfit ${d}(\phi ,\mathcal {X})\mathbf {1}_{\mathcal {T}^M}(t)$, occurs due to the limited number of product variants available under the MC technology. Oppositely, the AM technology enables customers to order products perfectly matching their desires, i.e., ${d}(\phi ,\mathcal {X})\mathbf {1}_{\mathcal {T}^M}(t)=0$ under AM. By this, the MC technology provides a higher customer willingness-to-pay penalization than the AM technology given that all other parameters are equal. Further, independently of the technology of the production, the utility is reduced in line with the *time disutility factor*, which is the product of the time sensitivity $\gamma (\tau )$ and the *normalized time lag* $\vert \tau -\frac{2t-1}{2}\vert /T$ between the product’s selling period and the customer’s ideal buying time $\tau$. Note that all the parameters may vary over the product life cycle (PLC), representing evolving customers’ interests and their sensitivity.

Overall, it is assumed that customers are rational utility maximizers (i.e., the customers choose the product variant that yields the maximum utility for them). Furthermore, the following assumptions are adopted about the customer purchasing behavior: given the selling price $p_t$, (i) a customer buys at most one product as soon as his or her utility exceeds the selling price, (ii) the customer buys at the first period *t* at which the previous purchasing condition is satisfied, if the product is available, and leaves the market. Assumption (i) implies that the initial market size *N* (the set of remaining potential customers after *t*) is denoted by $\Xi _t$. Our general notations and parametric assumptions for the decision variables, the HLB customer choice model, the technology characteristics, and the inventory policy (described in “Demand side: forecasting model” section) are summarized in Table 7 (see e-companion).

### Demand side: forecasting model

Let $D_{t}$ denote the demand at time $t$ and let $D_{j,t}$ specify the demand for the variant *j*. The demand for the mass-produced product $j$ with characteristics $x_j\in \mathcal {X}$ can be written as $D_{j,t} = \sum _{i \in \Xi _{t-1}} \mathbf {1}_{\{U^\mathcal {T}(\xi _i,t)>p_t\} \cap \{d(\phi _i,x_j)\le \delta \}}$, where $\delta =\frac{1}{2n}$ is the maximal level of tolerance between the desired product $\phi _i$ and its closest available analog $x_j$ in $\mathcal {X}$. Thus,

2 $$\begin{aligned} \left[ \begin{array}{lll} D_t&{}=&{}\sum _j\sum _{i \in \Xi _{t-1}} \mathbf {1}_{\{U^\mathcal {T}(\xi _i,t)>p_t\} \cap \{d(\phi _i,x_j)\le \delta \}}\text { if }\mathcal {T}(t)=MC\\ D_{t} &{}=&{} \sum _{i \in \Xi _{t-1}} \mathbf {1}_{\{U^\mathcal {T}(\xi _i,t)>p_t\}} \text { if }\mathcal {T}(t)=AM.\\ \end{array} \right. \nonumber \\ \end{aligned}$$

Note that the purchase happens if the individual customer’s utility is greater or equal to the price (see Timonina-Farkas, 2020) for an example with optimal purchase quantities). This goes in line with the well-known concept of *customer’s surplus*, which is greater than zero if the willingness-to-pay adjusted for the time and product misfit in our model (see Eq. (1)) exceeds the price. Clearly, the theoretical demand (2) depends on parameters which need to be estimated based on data. The quality of this estimation strongly influences the manufacturer’s decision about the production quantity (see Snyder and Shen 2019). This article proposes two methods to approximate the demand forecast $_*D'_{j,t}$ based on **censored** and **uncensored** information cases.

In the **censored** information case, the firm estimates the Bass distribution $F_{\tau }(t)$ and the demand forecast is obtained based on this estimate. A left subscript *c* (for *censored*) represents this alternative. Due to the independence of $\tau$ and $\phi$ for the population of size *N*, the demand forecast, thus, can be written as:3In the **uncensored** information case, the firm can statistically estimate the customer attributes $\omega , \lambda$ and $\gamma$ through market research. In this case, the demand forecast is computed for a given pricing strategy and is based only on the mean value of the demand in the customer choice model. This alternative is characterized by a left subscript *u* (for *uncensored*). The demand forecast yields4To approximate the demands, one can use the independence of $\tau$ and $\phi$ and the transfer theorem against the product law to compute $_ud'_{j,t}$:

5 $$\begin{aligned} _ud'_{j,t}= & {} \int _0^{T}\!\left( \int _{x_j-\frac{1}{2n}}^{x_j+\frac{1}{2n}}\left( \prod _{g=1}^{t-1}\mathbf {1}_{\{U ^\mathcal {T}(x,y,g)< p_g\}}\right) \right. \nonumber \\&\quad \left. \mathbf {1}_{\{U^\mathcal {T}(x,y,t)\ge p_t\}}f_\tau (x)dy\right) dx. \end{aligned}$$

Note that the desired and the produced products are considered to be close to each other, i.e., $d(\phi _i,x_j)\le \delta$, if and only if $\phi _i\in [x_j-\frac{1}{2n}, x_j+\frac{1}{2n}]$. Then, a change of variables $y'=x_{j'}-x_j+y$ in the above expression leads to the following equality under the MC technology: $_ud'_{j',t}=_ud'_{j,t},\; \forall j,j'$. The uncensored forecast alternative plays a central role in analytically grounding results of this paper (see Lemma 1 and Theorems 1, 2). Note that the following holds as a consequence of the uniform distribution in the Hotelling-Lancaster model, the independence of $\tau$ and $\phi$, and (3, 4):

6 $$\begin{aligned}&_{*}D'_{j,t}=_{*}\,D'_t/n,\; _{*}D'_{j,t}=N_{*}d'_{j,t},\; _{*}D'_t=N_{*}d'_t,\nonumber \\&_{*}d'_{t}=_{*}\,d'_{j,t}/n, \text { where }``{*}'' \text { represents } c \text { or }u. \end{aligned}$$

### Supply side: manufacturing policies

Consider the manufacturer, who can serially produce product variants using AM or/and MC technologies, characterized by the following assumptions:

1. AM and MC are both considered as flexible manufacturing systems – they can easily adapt to changes in the product variant and in the quantity being manufactured (Dong et al., 2020).
2. The lead time is zero, that is the product variants are produced instantaneously.
3. The product quality is similar under both AM and MC technologies. Nevertheless, AM is assumed to serve customers perfectly in terms of customer preferences $\phi$. Oppositely, the product assortment $\mathcal {X}$ is limited under MC.
4. Both technologies require one unit of common raw material to produce one product variant.
5. As the models of horizontal product differentiation assume no price discrimination, the selling prices and unit production costs are assumed to be identical for all variants at time $t$.
6. The manufacturing capacity per period $K^A$ under AM (respectively, $K^M$ under MC) is constant over the PLC, following Dong et al. (2020).
7. Following assumption (A6), we also assume that customer orders are served on the First-Come First-Served (FCFS) basis up until the capacity limit is reached.
8. AM follows the Make-To-Order (MTO) production process, i.e., the manufacturer does not hold inventory as products are tailor-made and shipped directly to customers. Producing ahead of time under AM would require prior knowledge of customer preferences, which is beyond the scope of this article. Differently, MC follows the Make-To-Stock (MTS) process with one-dimensional product customization (see the assumption of Jiang et al., 2006). As production capacity and the assortment size (set to $n_{max}$) are limited, the manufacturer may need to produce ahead of time to meet demand during the upcoming periods.

Given the product life cycle stage, the firm decides whether to switch from one production technology to another to maximize the profit while satisfying individual customer preferences. Following the setup of Lacroix et al. (2021), five production scenarios are analyzed. The scenarios are structured in line with the PLC curve and associated with product life cycle stages (i.e., introduction, growth, maturity and decline). Nevertheless, oppositely to the model presented in Lacroix et al. (2021), our article takes capacity constraints and inventory levels explicitly into account (see Shen et al., 2013). Overall, the following production scenarios are considered in this work:

**Base case (BC):** The manufacturer uses ***MC*** technology independently of the PLC stage;
**Case 1 (C1)**: ***AM ***$\rightarrow$ ***MC***: AM is used during the PLC introduction/growth stages;
**Case 2 (C2)**: ***MC ***$\rightarrow$ ***AM***: The manufacturer uses AM toward the PLC decline stage;
**Case 3 (C3)**: ***AM ***$\rightarrow$ ***MC*** $\rightarrow$ ***AM***: The manufacturer uses MC during the PLC maturity stage;
**Case 4 (C4)**: ***AM:*** The manufacturer uses only AM over the PLC.

Note that we do not consider scenarios with multiple technology switches during one stage of the PLC. This is due to the suboptimality of such scenarios in case no substantial change in demand is foreseen. Following our model setup, one can also eliminate the *MC *$\rightarrow$ ***AM*** $\rightarrow$ ***MC*** case because of higher costs associated with it at different stages of the PLC. The manufacturing technologies characteristics and notations are summarized in Table 1.

**Table 1:** MC and AM technology characteristics comparison

Characteristic	Production technology comparison	
	MC	AM
Production framework	MTS	MTO
Production period	$\mathcal {T}^M$	$\mathcal {T}^A$
Assortment size	$n \in [1;n_{max}]$	$n \in [1;+\infty [$
Unit production cost	$c^M(n)= c_B(1+(n-1)\delta )>0$	$c^A =$ constant $>0$
Setup cost	$k^M=$ constant $>0$	$k^A=$ constant $>0$
Production capacity	$K^{M}_j>0, \forall j \in \{1,\ldots ,n\}$	$K^A>0$, $K^A \le \sum _j K^{M}_j :=K^M$
Total production capacity	$K^M=\sum _j K^{M}_j=N\tilde{K}^M$	$K^A=N\tilde{K}^A$, $K^A \le K^M$
Holding cost	*h*	0 (no inventory under AM)
Salvage value	$v = 0.8 \times p_{T_{M\rightarrow A}}$	0 (no inventory under AM)

Under AM and MC technologies correspondingly, the firm incurs one-time fixed costs, $k^A(N)$, and $k^{M}(N)$, which are independent of the production quantity, though dependent on the market size, *N*. This reflects investment expenses on AM and MC equipment. The fixed cost $k^{M}(N)$ is counted once if $T_{A\rightarrow M}< T$, while the fixed cost $k^A(N)$ is incurred if $T_{A\rightarrow M}> 0$ or $T_{M\rightarrow A}< T$. Following the assumption of Dong et al. (2020), one can assume that $k_A(N) \ge k_M(N)$ since 3D-printers are typically more expensive than MC equipment. Based on the assumption of Lacroix et al. (2021), the authors set $k^A(N)=N\tilde{k}^A,\; k^M(N)=N\tilde{k}^M$, where $\tilde{k}^A=k^A/N$ and $\tilde{k}^M=k^M/N$. Further, per unit production costs are denoted by $c^A$ for AM technology and by $c^M(n)$ for MC technology, where $c^A>0$ and $c^M(n)>0$. Due to AM’s infinite flexibility in terms of product variants, $c^A$ does not depend on the product’s variety and is set constant for simplicity. By contrast, $c^M(n)= c_B(1+(n-1)\delta )$ depends on the number of mass-customized variants, where $c_B$ denotes a base cost and $\delta$ represents an incremental cost (following the form and notations in Dong et al. (2020)).

Besides the production framework (MTS and MTO), the product misfit penalty cost and the cost structure, AM and MC differ from each other in terms of production capacities (in line with Shen et al. (2013) and assumption (A6)). The total production capacity under MC is denoted by $K^M/n$ and computed per period and variant. Being equally distributed among the mass-customized variants, it is greater than the production capacity under AM per period, which is denoted by $K^A$. We set $K^A = \kappa \frac{N}{T} >0$, where $K^A \le \sum _j K^{M}_{j} :=K^M = \frac{\kappa }{\rho } \frac{N}{T}$, where $\kappa$ denotes the production capacity magnitude, and $\rho =\frac{K^A}{K^M}$ the production capacity ratio between AM and MC. To adapt the production capacities to the market size, they are set proportional to *N*. Proportionality coefficients introduced are $\tilde{K}^M=K^M/N=\kappa /(\rho T)$ and $\tilde{K}^A=K^A/N=\kappa /T$.

Overall, on the supply side, our article considers a capacity-constrained production that uses two flexible manufacturing systems, namely AM and MC. Three production capacity and inventory scenarios are investigaged: (i) the **MTO uncapacitated (MTOUC)** scenario (which serves as our reference case), where production capacities under AM and MC are assumed to be unlimited and the firm does not hold inventory; (ii) the **MTO capacitated (MTOC)** scenario, where the production capacities under AM and MC are constant over time, and the firm does not hold inventory; finally, (iii) the **MTS capacitated (MTSC)** scenario, which is similar to the MTOC scenario and allows the firm to hold inventory. The lead time is zero, that is, the variants are produced instantaneously. Also, the manufacturer can face a non-stationary demand for which the information distribution is not necessarily accessible. Therefore, in the MTSC scenario, the manufacturer carries an inventory for an assortment of mass-customized variants, sold to end-users. The inventory is controlled using an *adaptive inventory policy*, described in “Adaptive inventory policy” section.

Further, on the demand side of the MTOC scenario, the customers purchasing MC-produced goods are not able to observe the manufacturer’s inventory levels, do not make a second choice or return later in time if the first choice is unavailable due to production capacity shortage. In this case, unmet demand during the period is considered lost for the manufacturer, who incurs a stockout cost denoted by *s*. Further, the excess inventory is salvaged at value *v*, at the end of the last MC period, i.e., at $T_{M\rightarrow A}+1$. The salvage value corresponds to a fraction of the selling price of the last period under MC. The on-hand inventory is being held with a holding cost *h* per unit per period. The holding cost is assumed to be lower than the stockout cost, $h<s$, otherwise there would be no incentive for the firm to stock the variants. Also, to ensure the profitability of the manufacturer the following condition should hold: $p_t>v>c^M(n)$. The notations are summarized in Table 7 (see e-companion).

### Supply side: manufacturer’s profit

One can now compute the manufacturer’s profits implied by AM and MC technologies. We denote the profit at period $t$ by $\Pi ^\mathcal {T}_{t}(p)$. Based on the cost structures described in “Supply side: manufacturing policies” section, we formulate the following profit function for the AM technology:

7 $$\begin{aligned} \Pi ^\mathcal {T}_{t}(p)=(p_t-c_A) S_t-s L_t,\; \text {if}\;\mathcal {T}(t)=AM, \end{aligned}$$

where $S_t=\min \{D_t,K^A\}$ are the realized periodic sales and $L_t=\max \{0,D_t-K^A\}$ are the lost sales with unit cost $s$. Further, the profit function for MC technology can be defined as:

8 $$\begin{aligned}&\Pi ^\mathcal {T}_{t}(p) =\sum _j\big (p_t S_{j,t}-c_M(n) Q_{j,t}-h I_{j,t+1} \nonumber \\&\quad - s L_{j,t}+v I_{j,T_{M\rightarrow A}+1}\varvec{1}_{\{t=T_{M\rightarrow A}\}}\big ),\; \text {if}\;\mathcal {T}(t)=MC, \end{aligned}$$

where $S_{j,t}=\min \{D_{j,t},K^M_{j}+I_{j,t}\}$ are the the realized sales and $L_{j,t}=\max \{0,D_{j,t}-I_{j,t}-Q_{j,t}\}$ are the lost sales for the product $j$ at period $t$. Unlike the profit generated by AM technology, the profit under MC technology requires the optimization over the production quantity $Q_{j,t}$, as well as the knowledge about the inventory $I_{j,t+1}$ and the salvage amount $I_{j,T_{M\rightarrow A}+1}$ for product $j$. Thus, the manufacturer’s total profit, combining the functions (7) and (8), and incorporating the fixed costs for AM and MC technologies, can be written as:

9 $$\begin{aligned}&\Pi ^{^\mathcal {T}}(\{\xi _i\}^N_{i=1},p) = \sum _t\Pi ^\mathcal {T}_{t}(p)\nonumber \\&\quad -k^A(N) \varvec{1}_{\{t:\mathcal {T}(t)=AM\} \ne \emptyset }-k^M(N)\varvec{1}_{\{t:\mathcal {T}(t)=MC\}\ne \emptyset }. \end{aligned}$$

Note that the *additivity property* presented in the work of Lacroix et al. (2021) does not hold under the MTOC and MTSC scenarios in our profit function. Faced with production capacity constraints, the manufacturer risks that some potential buyers cannot be served due to product unavailability. For instance, if two customers have a positive utility and are interested in the same product variant, one customer will end up not purchasing if only one such variant is available. In general,

10 $$\begin{aligned} \Pi ^{^\mathcal {T}}(\{\xi _i\}^N_{i=1},p) \ne \sum _{i=1}^N\Pi ^{^\mathcal {T}}(\{\xi _i\},p). \end{aligned}$$

Thus, the individual customers’ profits cannot be defined independently of each other and the Law of Large Numbers (LLN) cannot be applied directly. Considering the *mean profit per customer* as the function $\pi ^\mathcal {T}(\{\xi _i\}^N_{i=1},p)$ defined as

11 $$\begin{aligned} \pi ^\mathcal {T}(\{\xi _i\}^N_{i=1},p)=\frac{1}{N}\Pi ^{\mathcal {T}}(\{\xi _i\}^N_{i=1},p), \end{aligned}$$

the main question which arises is if the limit in (11) exists and what its value might be. This question is addressed in “Supply side: mean profit per customer” section. In particular, the limit of (11) is described algorithmically and its almost sure (*a.s.*) convergence is proven. To further validate our optimization approach, the validity of the SAA approach is verified (see “SAA convergence” section).

### Supply side: mean profit per customer

As observed in (10), the arguments from Lacroix et al. (2021) to prove *a.s.* convergence in (11) to some limit can no longer be used. If such a limit existed, it would be the *theoretical mean profit per customer*, denoted by $\tilde{\pi }(\mathcal {T},n,p)$ for the given pricing, product variety and production strategies. In this section, a precise algorithmic formulation of $\tilde{\pi }(\mathcal {T},n,p)$ is developed and the aforementioned *a.s.* convergence is proven. Note that, for the $_ud'_t$, we have an integral form (5):

12 $$\begin{aligned}&_u d' _t=\int _0^{T}\!\left( \int _0^1\left( \prod _{g=1}^{t-1}\mathbf {1}_{\{U^\mathcal {T}(x,y,g)<p_g\}}\right) \right. \nonumber \\&\quad \left. \mathbf {1}_{\{U^{\mathcal {T}}(x,y,t)\ge p_t\}}f_\tau (x)dy\right) dx. \end{aligned}$$

#### Lemma 1

The actual mean demand convergences almost surely (*a.s.*) to the uncensored mean demand for all *t*, *j*.

#### Proof of Lemma 1

By the Law of Large Numbers (LLN) the *a.s.* convergence of the actual mean demand to the uncensored mean demand for all *t*, *j* is obtained. From this follows the *a.s.* convergence of the quantities involved in the profit function, that is the mean of target and on-hand inventory levels, production quantity, sales and lost sales, on-hand inventory for the next period and, finally, the profit per period (see e-companion EC. 2 for more details).

The limiting total target inventory level per period $(i'_1,\ldots ,i'_{T+1})$ can be obtained from $(_*d'_1,\ldots ,_*d'_{t})$ by an algorithm that resembles the method (WFS), described in Section 4.1. Using this key observation and Lemma  1, the *theoretical mean profit per customer* and its algorithmic formulation can now be formally defined. .

$$\square $$

#### Theorem 1

**Mean profit per customer,** ***a.s.*convergence.** If $(\xi _i)_{1\le i\le N}$ are i.i.d. random variables, then

13 $$\begin{aligned}&\Pi ^{^\mathcal {T}}(\{\xi _i\}^N_{i=1},p)\bigm /N\rightarrow _{a.s.}\tilde{\pi }(\mathcal {T},n,p),\; \text {where} \end{aligned}$$

14 $$\begin{aligned}&\tilde{\pi }(\mathcal {T},n,p)=\pi ^\mathcal {T}_1+\ldots +\pi ^\mathcal {T}_T-\tilde{k}^A\varvec{1}_{\{T_{A\rightarrow M}>0\text { or }T_{M\rightarrow A}<T\}}\nonumber \\&\quad -\tilde{k}^M\varvec{1}_{\{T_{A\rightarrow M}<T\}}, \end{aligned}$$

and the $\pi ^\mathcal {T}_t$’s are obtained from algorithm (A-MTS) (see e-companion EC.4).

#### Proof of Theorem 1

The proof follows from Lemma 1 (see e-companion EC.2).

$$\square $$

Further, the following corollaries arise using arguments similar to those detailed in Lemma 1 and Theorem 1. They provide the basis for algorithmic computations of the theoretical mean profits per customer derived from the equation for $_ud'_t$, in the MTOC and MTOUC scenarios.

#### Corollary 1

**Mean profit per customer – the MTOC scenario.** The *a.s.* convergence holds in the MTOC setup, while the limit of the *mean profit per customer* can be obtained by a simplified version of the (A-MTS) algorithm (see e-companion EC.5).

#### Corollary 2

**Mean profit per customer – the MTOUC scenario.** In the absence of inventory and production capacity limitation, the case of Lacroix et al. (2021) is recovered, for which the *a.s.* convergence of the mean profit is derived (see e-companion EC.6).

## Solution approach to maximize profit

The manufacturer aims to maximize the total expected profit by jointly deciding on (i) the technology-switching times $(T_{A\rightarrow M},T_{M\rightarrow A})$, (ii) the pricing strategy $p_t$, (iii) the product variety *n*, and (iv) the production quantity $Q_t$ under MC. Our optimization problem is formulated using the *theoretical mean profit per customer* in line with Theorem 1:

15 $$\begin{aligned} \begin{aligned}&\pi ^*:=\underset{\begin{array}{c} \mathcal {T} \\ 1\le n\le n_{max}\\ \end{array}}{\text {max}}\; \underset{\begin{array}{c} p\in \mathcal {P} \end{array}}{\text {max}} \quad \tilde{\pi }(\mathcal {T},n,p). \end{aligned} \end{aligned}$$

Importantly, as demonstrated in “Adaptive inventory policy” section, the optimal production quantity can be written in a closed-form and, thus, one does not need to specify this decision variable in the formulation (15) for a numerical solution. Furthermore, pricing decisions are separable from the decisions about switching times and product variety. Thus, let us first consider the inner maximization, which is a non-convex optimization problem:

16 $$\begin{aligned} \tilde{\pi }(\mathcal {T},n):=\max _{p\in \mathcal {P}}\tilde{\pi }(\mathcal {T},n,p). \end{aligned}$$

Its solution can be obtained via methods similar to those described in Lacroix et al. (2021), i.e., via the use of the SAA framework (Shapiro et al., 2014) and a direct local search method, in particular a Pattern Search (PS). Note that the PS heuristic is commonly used for nonlinear programming problems with discontinuous non-smooth objectives (Chinneck, 2015).

In the outer optimization, the optimal value $\tilde{\pi }(\mathcal {T},n,p)$ is estimated based on Theorem 1, Corollaries 1, 2 and employing algorithms (wfs, A-MTS, A-MTOC, A-MTOUC). As the computation of the value $_ud'_t$ in Eq. (4) is based on *T* integrals (12) and is computationally complex, the function (15) is optimized using the SAA framework. For this, one estimates the maximum mean profit per customer (16) and finds the optimal pricing strategy for a fixed production and a product variety strategy $\mathcal {T}$ and $n$, correspondingly. Multiple estimates are generated to efficiently obtain the value $\pi ^*$ which guarantees a lower bound on the profit via associated strategies $p^*$, $\mathcal {T}^*$, $n^*$.

Importantly, one can prove that the SAA convergence holds even in the absence of the additivity property (10). A robustness test is conducted in “Robustness test and population sample size choice” section to determine a sufficient sample population size for the SAA optimization problem.

### Adaptive inventory policy

The optimal quantity $Q_{j,t}$ for the mass-produced variant $j$ must correspond to the minimum between the capacity $K^M_j$ and the demand $D_{j,t}$ adjusted for the discrepancy between *available* and *necessary* (i.e., *target*) inventories on stock. The inventory on stock must suffice for a part of the present demand and a part of future demand in case the production capacity is low. Denote the on-hand inventory by $I_{j,t}$ and note that it might differ from the *target* inventory $I'_{j,t}$, which is necessary to hold in reality already at time $t$ to avoid losses because of upcoming demands. The production quantities and the evolution of inventories in time can be written as:

17 $$\begin{aligned}&Q_{j,t}=\varvec{1}_{\{\mathcal {T}(t)=MC\}}\min \{K^M_j, \max \{0,I'_{j,t}-I_{j,t}+D_{j,t}\}\},\nonumber \\&\quad Q_t=\sum _j Q_{j,t} \end{aligned}$$

18 $$\begin{aligned}&I_{j,t+1}=\max \{0,I_{j,t}+Q_{j,t}-D_{j,t}\},\;I_{t+1}=\sum _j I_{j,t+1}.\nonumber \\ \end{aligned}$$

**Fig. 2:**
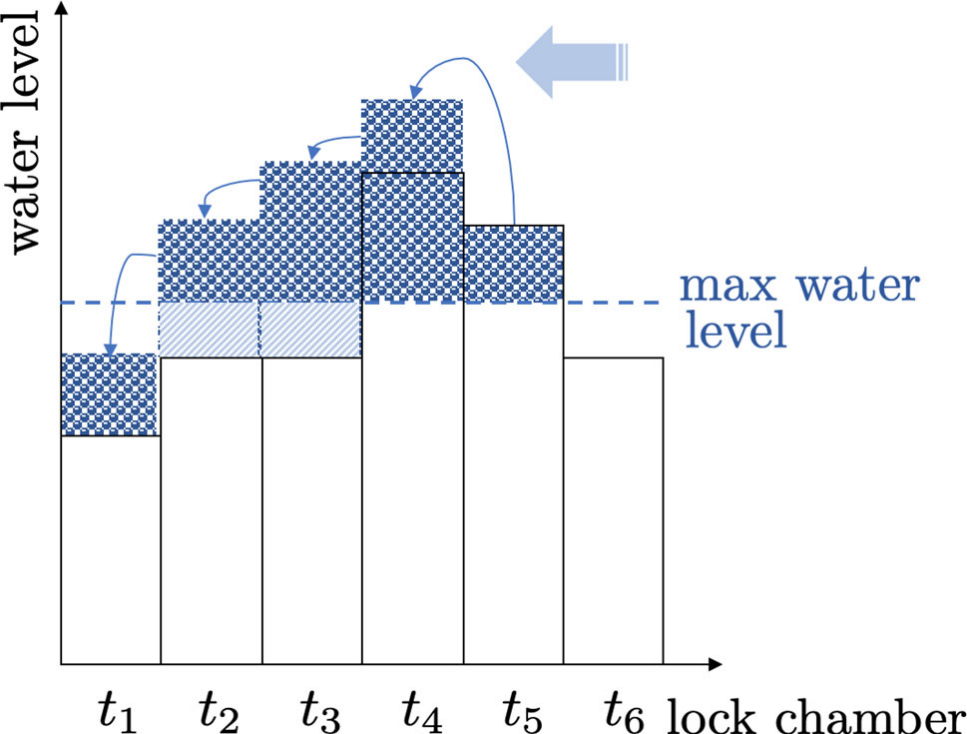
Water filling scheme analogy

Clearly, one would wish for no discrepancy between the available and target inventories $I_{j,t}$ and $I'_{j,t}$. To reduce this discrepancy, an *adaptive inventory policy* is developed, which relies on a procedure described below and accounts for the fact that the demand forecast is interdependent across the PLC due to the influence of production capacity on the customer’s ability to purchase. The on-hand inventory $I_{j,t}$ can be monitored periodically at the beginning of each period $t$ and one can assume, without loss of generality, that there are no initial inventories (i.e., $I_{j,T_{A \rightarrow M +1}} = 0$):

- **Backward step:** The *backward step* is based on demand forecasts $_{*}D'_{j,t}$ for each period (see (3) and (4)). Going backward in time (i.e., starting at $t=T$), the manufacturer estimates the target inventory $I'_{j,t}$ for each period $t$ and variant $j$. For this, the manufacturer observes if the demand forecast exceeds the MC production capacity. In case of an excess demand, the amount should be present in stock already in period $t-1$. Repeating the operation, the manufacturer decides about inventories up to period $t=1$. Note that by this the manufacturer employs the *“Water Filling Scheme”* (WFS) which is an algorithm typically used in information theory (Yu and Cioffi, 2001), providing equalization strategies on communications channels. Indeed, if the water level (i.e., demand forecast) in the lock chamber (i.e., period *t*) exceeds the maximum allowed water level (i.e., MC production capacity), the valve separating the current lock chamber from the previous one (i.e., period $t-1$) needs to be open (see Fig. 2 for analogy). Denoting the mean target inventory level per period and variant by $i'_{j,t}$, $1\le j\le n,\; 1\le t\le T+1$, the total target inventory level per period by $I'_{t}$, $1\le t\le T+1$ and the mean total target inventory level per period by $i'_{t}$, $1\le t\le T+1$, the following algorithm is derived:
  $$\begin{aligned} \begin{array}{c} \boxed {\mathtt {(I'_1,\ldots ,I'_{T+1})=WFS(\mathcal {T},n,(_{*}D'_1,\ldots ,_{*}D'_T)):}} \end{array} \end{aligned}$$
  WFS $$\begin{aligned} \begin{array}{ll} t=T,\;I'_{T+1}=i'_{T+1}=I'_{j,T+1}=i'_{j,T+1}=0\\ \mathtt {while }t>0\\ \qquad \mathtt {if }\, t>T_{A\rightarrow M}\\ \qquad \qquad I'_{j,t}=\max ((_{*}D'_{j,t}-K^M_j)\mathbf {1}_{\{\mathcal {T}(t)=MC\}}+I'_{j,t+1},0)\\ \qquad \qquad i'_{j,t}=\frac{1}{N}I'_{j,t}=\max ((_{*}d'_{j,t}-\tilde{K}^M/n)\mathbf {1}_{\{\mathcal {T}(t)=MC\}}+i'_{j,t+1},0)\\ \qquad \qquad I'_{t}=\sum _jI'_{j,t}\\ \qquad \qquad i'_{t}=\sum _ji'_{j,t}=I'_t/N\\ \qquad \qquad t=t-1\\ \qquad \mathtt {else\, if }\le T_{A\rightarrow M}\\ \qquad \qquad i'_t=0\\ \qquad \qquad t=t-1\\ \qquad \mathtt {end}\\ \mathtt {end}\\ \end{array} \end{aligned}$$
  Note that the algorithm is proposed for computation of inventory levels under MC technology, as soon as the AM technology does not require holding on-hand stock, i.e., $I'_{t}=i'_{t}=I'_{j,t}=i'_{j,t}=0,\; \text {if}\; \mathcal {T}(t)=AM$.
- **Forward step:** The forward determines the optimal production quantity $Q_{j,t}$ after observing the actual demand $D_{j,t}$. By this, the discrepancy between the on-hand inventory level $I_{j,t}$ and the target inventory $I'_{j,t}$ computed in the *backward step* is reduced. Going forward across time periods (i.e., starting at $t=1$), one determines the production quantity and the inventory amount using closed-form solutions (17) and (18), correspondingly. The starting inventory levels at $t=1$ are set at zero, i.e., $I_{j,1}=0,\;\forall j$. The mean inventories amounts can be computed as $i_{j,t}=I_{j,t}/N$, $i_t=I_t/N$.

### SAA convergence

As demonstrated in “Supply side: manufacturer’s profit” section the objective function is not equal to the sum of profits per customer. Under capacity constraints, the demand becomes interdependent across the PLC. Nevertheless, Lemma  1 (see EC.2) and Theorem 1 prove that the mean profit per customer can be derived algorithmically from the integrals defining the value of $_ud'_t$ (4). Also, the *a.s.* convergence of quantities discussed in Lemma 1 depends on that of the ratios $D_t/N$ and $D_{j,t}/N$. The SAA approach purely relies on the existence of a *p*-uniform a.s. convergence (UASC) (Shapiro et al., 2014).

**Fig. 3:**
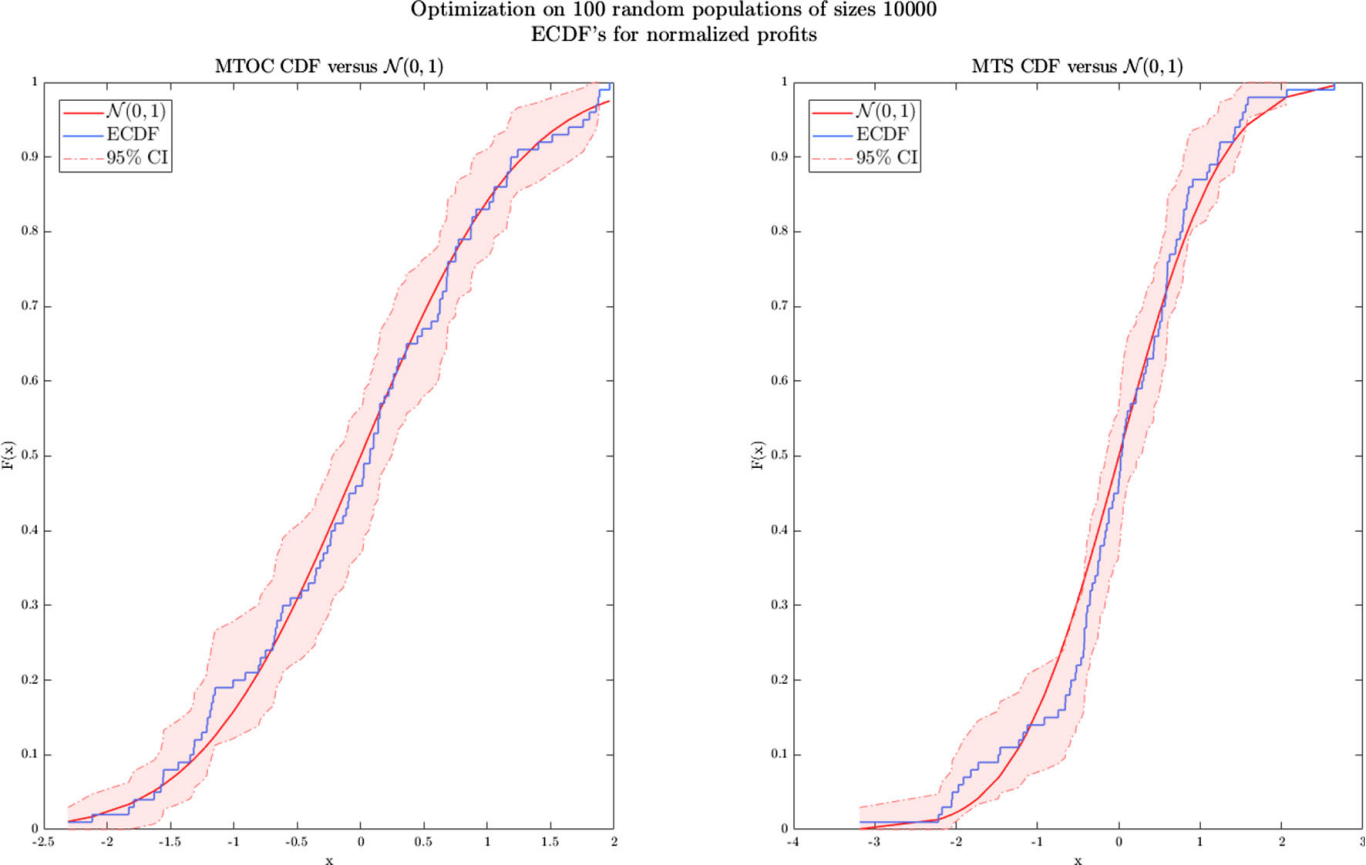
SAA validation

#### Lemma 2

**Uniform a.s. convergence of the ratio** $\mathbf {D_{j,t}/N}$. If the convergence in Lemma 1 holds for ratios $D_t/N$ and $D_{j,t}/N$, then

UASC $$\begin{aligned} \pi ^\mathcal {T}(\{\xi _i\}^N_{i=1},p)\displaystyle {\longrightarrow _{a.s.}}\;\tilde{\pi }(\mathcal {T},n,p)\;\text {uniformly in } p\in \mathcal {P} \end{aligned}$$

where $$\mathcal {P}=[0,\max _{x\in [0,T]} \omega (x)]^T$$.

Note that one can prove the convergence of ratios $D_{t}/N$ and $D_{j,t}/N$ using the same argument as in ((Lacroix et al., 2021),Theorem 1).

#### Theorem 2

**SAA convergence of the mean profits.** If $(\xi _i)_{i\ge 1}$ are i.i.d. random variables and if

H $$\begin{aligned} \mu _\omega<\!\!<\mu _L;\text { and } \forall c,t,\;\mu _L\left( \delta (\cdot ,t)^{-1}(\{c\})\right) =0, \end{aligned}$$

then the statement (UASC) holds for ratios $D_{j,t}/N$ and $D_{j,t}/N$. As a consequence of Lemma 2, if

$$\begin{aligned} \left\{ \begin{array}{l} p^{(*,N)}\text { achieves }\max _{p\in \mathcal {P}}\pi ^\mathcal {T}(\{\xi _i\}^N_{i=1},p):=\pi ^{(*,\mathcal {T})}(\{\xi _i\}^N_{i=1},p^{(*,N)}),\\ p^{*}\text { achieves }\max _{p\in \mathcal {P}} \tilde{\pi }(\mathcal {T},n,p)\;(=\tilde{\pi }(\mathcal {T},n)=\tilde{\pi }(\mathcal {T},n,p^*)),\\ \end{array} \right. \nonumber \\ \end{aligned}$$

then

SAA $$\begin{aligned} \left\{ \begin{array}{l} p^{(*,N)}\rightarrow p^*;\\ \pi ^{(*,\mathcal {T})}(\{\xi _i\}^N_{i=1},p^{(*,N)})\rightarrow _{a.s.}\tilde{\pi }(\mathcal {T},n). \\ \end{array} \right. \end{aligned}$$

#### Proof of Theorem 2

See EC.3. $$\square $$

## Numerical experiments

Numerical experiments using the PS algorithm are carried out to highlight the benefits of and required conditions for interchanging capacitated AM and MC over the PLC. Specifically, capacity constraints and inventory decisions are explored with the aim of better understanding their effects on this new manufacturing approach.

### Robustness test and population sample size choice

In this section, 100 optimization strategies for 100 independent sample population paths of size 10,000 are tested in order to assess if the sample population of 10,000 is sufficient for approximating $p^*,\pi ^*,\mathcal {T}^*,n^*$. Then, variations on the obtained optimal quantities are evaluated and the mean profit for each sampled population and optimization strategies is maximized. The profit is computed from materialized sales. Figure 3 illustrates and validates the robustness by comparing the normalized mean profits with the normal distribution, in the MTOC and MTS cases. Very low standard deviations for the mean optimal profits are observed.

**Table 2:** SAA mean profit variations, sample size $10^4$

Statistical estimators	MTOC	MTS
mean(mean profits)	5.79	5.91
std(mean profits)	0.025	0.027

### Sensitivity analysis

Next, sensitivity analyses are performed and several parametric scenarios issued from our model are investigated (e.g., “Value of holding inventory” and “Value of pricing flexibility” sections). Our parametric setup is similar to ((Lacroix et al., 2021),Table 4), while capacity and inventory parameters are set as follows: the fixed production capacity magnitude and ratio are $\kappa = 0.5$ and $\rho = K^A/K^M=0.5$ respectively; the holding cost is $h=0.5$; the stockout cost is $s=0.8$ per unit of unsatisfied demand; the potential remaining inventory at the end of MC period is salvaged at $v=0.8 p_{T_{M \rightarrow A}}$. Table 3 reports the baseline parameters.

**Table 3:** Baseline parameter values

Parameter	*p*	*q*	*N*	*T*	$n_{max}$	$k_M$	$k_A$	$\delta$
Value	0.02	0.6	10,000	12	15	100	150% of $k_M$	0.06
	$$c_b $$	$$c_M$$	$$c_A$$	$$\kappa $$	$$\rho $$	*h*	*s*	*v*
	2	2.48	180% of $c_b$	0.5	$\frac{K^A}{K^M}=0.5$	0.5	0.8	$0.8 \times p_{T_{M \rightarrow A}}$

***Production Capacity—Ratios and Magnitudes—*** ***Sensitivity*** While analyzing several production capacity ratios between AM and MC, namely $\frac{K^M}{K^A}\in \{10;4;2;1.33;1\}$, the production capacity under AM technology is considered as fixed and the total production capacity under MC technology is being varied. The assumption (A6) which implies $K^A \le K^M$ is used, while *low, medium,* and *high* production magnitudes are analyzed for each production capacity ratio. In particular, the capacity levels $K^A \in \{41; 208; 416\}$ are considered and the values for $K^M$ are determined through the production capacity ratios.

**Fig. 4:**
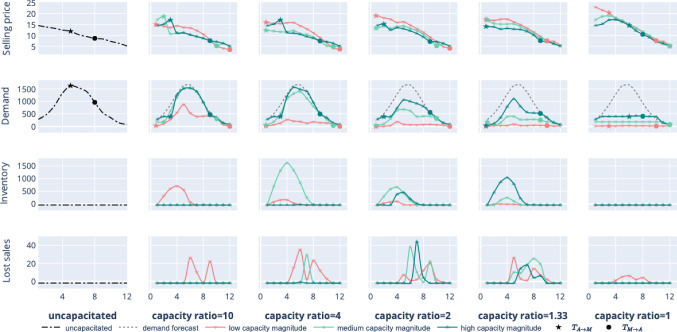
Sensitivity analysis of production capacity ratios and magnitude under AM and MC technologies, in the MTS scenario

Firstly, Fig. 4 demonstrates the behavior of the optimal pricing strategy $p^*$. One can observe that the selling price is not monotonically decreasing as it would be in the uncapacitated case (Lacroix et al., 2021). Instead, it exhibits an increasing-decreasing pattern when the demand tends toward the production capacity under AM and MC. Thus, to avoid potential lost sales from capacity shortage, the firm charges high upfront prices by an increasing pricing policy. This strategy helps the firm to boost short-term profits from the most eager and interested initial customers. Compared with the case with *high* production magnitude, the firm charges higher selling prices during the products’ introduction for the *low* and *medium* capacity magnitudes. This is because the firm can offer fewer products due to the capacity constraint, and, consequently, tries to attract fewer customers but those with higher product valuation. This also leads to higher profit and reduced lost sales. Furthermore, the effect is strengthened due to the forward-looking behavior of customers since they have a decreasing willingness-to-pay (see (1)).

Secondly, Fig. 4 shows the demand process $D_t$. As customers are modeled through a utility-based demand, the selling price has a direct impact on the demand diffusion pattern. In the MTOUC case, one recovers the traditional bell-shaped curve of the demand. In the MTS case, the demand patterns and demand forecast are also bell-shaped for *high* capacity magnitudes under MC (see demand for *medium* and *high* capacity magnitudes and $\frac{K^M}{K^A}=10$). As the production capacity ratio diminishes, the demand trajectory tends to flatten. At the beginning and the end of the PLC, there are fewer customers due to their ideal buying time distribution. Less product quantity is sufficient to meet the demand. The demand grows and the firm can sell at full production capacity toward the middle of the PLC.

Thirdly, Fig. 4 depicts the on-hand inventories to satisfy the demand. For low capacities, the inventory decreases as the production capacity ratio diminishes. This is due to opposite trends of the selling price and the demand patterns. Next, one can observe that the lost sales are negligible. Also, for similar production capacities and as the production capacity magnitude increases, AM technology is used more often by the manufacturer. Figure 4 illustrates this through the technology-switching times. However, for the low capacity magnitude case, and when $K^A$ is much lower than $K^M$, the manufacturer does not switch to AM (see the technology-switching times for $\frac{K^M}{K^A}=10$). During the introduction and decline stages of the PLC, higher production capacity magnitudes allow the firm to offset the higher fixed and production costs of AM compared with those of MC. Overall, the manufacturer benefits from adopting AM at the beginning and the end of the PLC in the MTS scenario. Switching to MC in the middle of the product life cycle could be profitable provided a high production capacity magnitude and similar capacities under AM and MC.

**Fig. 5:**
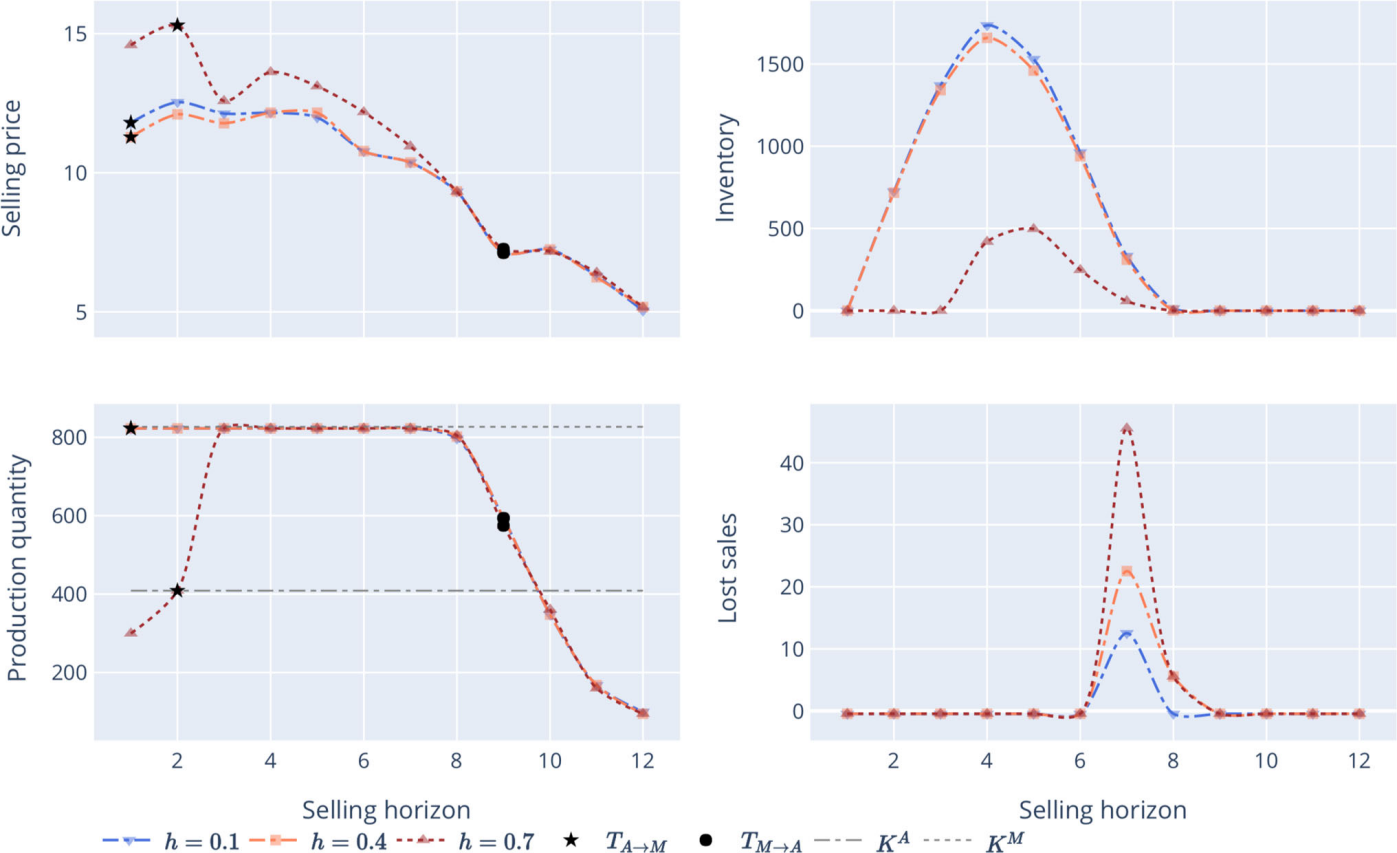
Holding cost sensitivity

***Holding Cost Sensitivity*** The sensitivity of our results to the holding cost and its impact on profits are examined in the MTSC scenario. Figure 5 shows that the firm charges a higher selling price as the holding cost value increases.

**Table 4:** Holding cost impact on the mean profit per customer

Production strategy	Mean profit per customer for each holding cost value		
	${\textbf {h}}=0.1$	${\textbf {h}}=0.4$	${\textbf {h}}=0.7$
*MC*	6.06	5.85	5.66
$MC \rightarrow AM$	**6.12**	**5.93**	5.68
$AM \rightarrow MC$	5.91	5.82	5.74
*AM*	4.02	3.81	4.04
$AM \rightarrow MC \rightarrow AM$	5.99	5.92	**5.88**

The numbers in bold serve to highlight the highest mean profit per customer for each holding cost value

If the holding cost is lower, the firm decides to stock more inventory to satisfy the demand and, oppositely, to stock less when it is more expensive. Moreover, it is more beneficial to start producing with AM instead of MC when the holding cost is high, and when the production capacity of this technology allows to meet the demand. Table 4 reports the holding cost impact on the technology-switching scenario and on the *mean profit per customer*. As expected, a lower holding cost yields a higher mean profit per customer and as the holding cost becomes expensive it is beneficial to start producing with AM at the beginning of the PLC when there are fewer but more excited customers (i.e., with a higher utility).

### Value of holding inventory

To identify the value of holding inventory across the PLC, three capacity and inventory scenarios are examined, namely MTOUC, MTOC and MTSC. For each of these scenarios, one extracts the optimal mean profit per customer for every production strategy and highlights the highest among them, which is the AM$\rightarrow$MC$\rightarrow$AM strategy for all the reference cases (see Table 5). Considering the MTOUC case as a benchmark, Table 5 shows that capacity constraints generate a 24% profit loss under the MTOC scenario, and a 22% profit loss under the MTSC scenario. Nevertheless, adopting an $AM \rightarrow MC \rightarrow AM$ technology-switching strategy allows 17.6%, 12.6%, 1.9% profit gains compared to the MC strategy for MTOUC, MTOC and MTSC scenarios correspondingly.

**Table 5:** Capacity and inventory strategies impact on the mean profit per customer

Production strategy	Mean profit per customer		
	**MTOUC**	**MTOC**	**MTSC**
*MC*	6.42	5.08	5.79
$MC \rightarrow AM$	7.22	5.16	5.87
$AM \rightarrow MC$	7.46	5.62	5.80
*AM*	7.48	4.05	4.06
$AM \rightarrow MC \rightarrow AM$	**7.55**	**5.72**	**5.90**

Taking the recent phenomenon of Omega $\times$ Swatch MoonSwatches as an example (see Financial Times review of Foulkes (2022)) and assuming that the company’s production costs were aligned with our parameters, one can observe that the mean profit per Swatch customer could have been increased by 16.1% if the $AM \rightarrow MC \rightarrow AM$ strategy had been adopted and holding inventory had been allowed, i.e., if MTSC had been implemented instead of Swatch’s MTOC scenario, which clearly led the company to encounter difficulties with addressing the unexpectedly high demand.

**Fig. 6:**
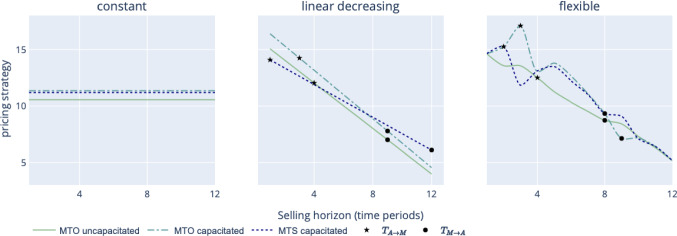
Selling price trajectory for each production and inventory scenario

### Value of pricing flexibility

To investigate the impact of pricing flexibility on the manufacturer’s expected mean profit per customer, numerical experiments are performed and three selling price trajectories are analyzed, i.e., constant, linear decreasing, and flexible.

In line with our findings in “Sensitivity analysis” section, Fig. 6 reveals that it is optimal to charge more under MTOC and MTSC scenarios if selling prices must stay constant over the time horizon. In contrast, when the pricing pattern is linear decreasing, the firm charges the lowest price under the MTSC case and the highest price under the MTOC case up to the middle of the PLC. Following the example of the Omega $\times$ Swatch MoonSwatches, whose selling price is 260,- USD per watch in comparison to 6600,- USD for an Omega Speedmaster, and irrespective of the pricing strategy the company implements, one could assume that the customers’ willingness-to-pay and, thus, the initial selling price was underestimated and chosen suboptimally, which, in turn, might have initiated the phenomenal demand for the proposed eleven MoonSwatch variants even though the full product variability as under AM technology was not proposed. Given flexibility in their pricing strategy, the optimal solution for Swatch would be to increase prices for MoonSwatches in the short-run (see flexible pricing strategy in Fig. 6 with a peak in selling price under the MTOC case).

Although MTOUC and MTOC scenarios only employ AM during the whole PLC, the MTSC scenario uses MC technology on its own. To offset the product misfit penalty cost only incurred under MC and to attract more customers, the firm relies on lower upfront prices until the demand peak. The flexible pricing strategy results in an optimal non-convex path. Interestingly, one observes “reversed” selling prices set at $t=3$ and $t=9$ in our flexible pricing trajectories. This can be explained by technology-switching and production capacity effects on the selling price, and, consequently, on demand. AM is used up to period $t=3$ in the MTOC scenario whereas it is used until period $t=2$ in the MTSC scenario. The price is thus set higher for the third period under AM in the MTOC scenario. In both the MTOC and the MTSC scenarios, when the firm switches to AM at the end of the PLC, the selling price first increases before decreasing as demand is close to the production capacity under AM. The relative gain of the flexible versus the constant pricing strategy is defined as $(\pi ^*_{flex} - \pi ^*_{const})\cdot (\pi ^*_{const})^{-1}$ and, similarly, the flexible versus linear decreasing pricing strategy is defined as $(\pi ^*_{flex} - \pi ^*_{dec})\cdot (\pi ^*_{dec})^{-1}$. Table 6 summarizes the results and shows that there is a significant gap between a constant and a flexible pricing policy.

**Table 6:** Impact of the pricing trajectory on profit and technology-switching scenario

*Pricing trajectory*	MTOUC			
	$\pi ^{*}(\$)$	$T_{A \rightarrow M}$	$T_{M \rightarrow A}$	Production strategy
Constant	6.19	3	6	$AM \rightarrow MC \rightarrow AM$
Linear decreasing	7.44	12	13	*AM*
Flexible	7.55	4	8	$AM \rightarrow MC \rightarrow AM$
MTOC				
Constant	4.11	2	7	$AM \rightarrow MC \rightarrow AM$
Linear decreasing	5.14	12	13	*AM*
Flexible	5.73	3	9	$AM \rightarrow MC \rightarrow AM$
MTSC				
Constant	5.04	0	7	$MC \rightarrow AM$
Linear decreasing	5.65	0	12	*MC*
Flexible	5.86	2	8	$AM \rightarrow MC \rightarrow AM$

Note that the profit gap between the flexible and decreasing pricing policies is small when the firm has an infinite capacity (i.e., +1.5% for the MTOUC case). On that basis, it might be more cost-effective to apply a simple decreasing pricing policy as suggested by the marketing literature. It works well in practice and can avoid unobserved fees. Under capacity constraints, the gap strongly increases and benefits the flexible pricing strategy (+11.5% in the MTOC scenario). Thus, the firm is better off using a flexible pricing policy displaying an increasing-decreasing pattern. The ability to increase prices during the PLC helps the manufacturer to better align supply and demand (e.g., to cool down the demand for products of the Omega $\times$ Swatch collaboration).

Our results are consistent with those of Shen et al. (2013), who report that an increasing-decreasing pricing strategy, combined with optimal production/inventory policies, is profitable under capacity constraints. The value of pricing flexibility is highest (+11.5%) under the MTOC scenario, and decreases when the firm carries inventory (+3.7%). Holding inventory might lessen the need for pricing flexibility. In other words, holding inventory allows the firm to have higher profits by itself.

These findings can help operations managers to better understand this new manufacturing approach and to take advantage of the potential marketing and operations benefits by combining AM with MC technology. Facing forward-looking customers and their individual preferences, adopting AM, in combination with MC, under capacity constraints, could improve a manufacturer’s profit if the production capacity magnitude is high enough and close to that of MC technology.

## Conclusion and managerial insights

This paper investigates the conditions under which a capacity-constrained monopolist manufacturer could combine the benefits of AM technology for product customization with the traditional MC technology to increase profits. Given the stage of the PLC, the firm jointly decides on marketing (pricing policy, product variety) and on operations (technology-switching times, production quantity, inventory) strategies to maximize profit while addressing individual customer preferences. Our model positions itself at the marketing-operations interface. It considers not only the supply side with the technology choice in a dynamic setting across the PLC, but also the demand side to account for customer heterogeneity and their forward-looking behavior. By this, our work provides an innovative methodology to leverage customer-centricity and optimize operations and marketing strategies.

First, the article investigates several technology-switching scenarios under different production capacity and inventory cases. In the scenario where the firm holds inventory under MC, a customer-centric *adaptive inventory policy* is developed with an aim to address an interdependent non-stationary demand. The inventory policy is built on a so-called *water filling algorithm*, which is typically used in information theory but can be adapted to fit our manufacturing context. Such inventory policy allows us to specify a closed-form solution for the production quantity decision. Formalizing the resulting non-convex-concave optimization problem, our work exploits the analytical properties of the demand forecast and derives an algorithmic formulation for the objective function under capacity and inventory scenarios. The optimization problem is solved using the SAA framework, while robustness tests are performed to check the convergence of the approximation given the population sample size used in our numerical experiments.

Our numerical results demonstrate that the combination of customer-centric strategies with the new usage of AM and MC could benefit a manufacturer. In particular, on the operations side, significant profit improvements could be achieved with an AM–MC–AM technology-switching scenario if certain capacity and inventory conditions hold: in particular, sufficient production capacities but high holding costs under MC can lead to the profitability of the switching strategy.

Our findings also demonstrate the benefits of pricing flexibility, which are the highest when capacity is limited and when the firm does not hold inventory. This gives an insight into a possible policy for addressing the recent phenomenon of Omega $\times$ Swatch MoonSwatches, which are produced by Swatch at a capacity limit without holding inventory but cannot satisfy the increasing demand. If the firm would have an option to hold inventory, facing *low* or *medium* production capacity magnitudes would imply both increasing the selling price and to stock more. This strategy would help both in preventing lost sales and meeting the demand peak during the growth stage of the PLC.

Although there are limitations due to the lack of real-world data availability, we believe that our work sheds light on this new manufacturing opportunity. Finally, the manufacturing approaches showcased in this article could be implemented by decision-makers to leverage customer-centricity whilst benefiting from the novel technology-switching practice, which operates an Industry 4.0 technology such as AM for product customization.

## EC.1. Notations and parametric assumptions

See Table 7.

**Table 7:** Notations and parametric assumptions

Parameters		Assumptions
*N*	: Initial market size of potential adopters	$N \in \mathbb {N}$
$\Xi _t$	: Remaining potential adopters at period *t*	$\big \{\cup ^{T}_{m=t} \Xi _m\big \}^T_{t=1}$
*T*	: Length of the finite selling horizon	$T \in \mathbb {N}$
*t*	: Subscript denoting period	$\{t\}^{T}_{t=1}$
$\mathcal {T}$	: Set of production strategies characterized by $(T_{A \rightarrow M}, T_{M \rightarrow A})$	$\mathcal {T} \in \{T_{A\rightarrow M},T_{M\rightarrow A}\}^{T}$
$T_{A \rightarrow M}$	: Technology-switching time when the manufacturer switches from AM to MC	$0 \le T_{A\rightarrow M}< T_{M\rightarrow A}$
$T_{M \rightarrow A}$	: Technology-switching time when the manufacturer switches from MC to AM	$T_{A\rightarrow M}< T_{M\rightarrow A}< T+1$
$\Phi$	: Virtual space of horizontally differentiated products	$\Phi =[0,1]$
$\phi$	: Customer’s ideal product variant	$\mathbb {P}_\phi =\mathcal {U}([0,1])$
$\tau$	: Customer’s ideal buying time	see Lacroix et al. (2021)
*p*, *q*	: Bass innovation and imitation coefficients, respectively	$p,q \in \mathbb {R}^{+}$
$\xi$	: Random customer characterized by $\tau$ and $\phi$	$\xi =(\tau ,\phi )$ with $\mathbb {P}_\xi =\mathbb {P}_\tau \varnothing$
*n*	: Number of mass-customized variants to offer to customers under MC	$1\le n\le n_{max}$
$\mathcal {X}$	: Set of mass-customized product variants offered under MC	$\mathcal {X}=\{x_1,\ldots ,x_n\}\subset [0,1]^n$
*j*	: Subscript denoting the mass-customized variant	$j \in \{1,\ldots ,n\}$
$x_j$	: Location of product variant *j* on the virtual product space	$\forall j \in \{1,\ldots ,n\}$
$w_t(\tau )$	: Customer’s willingness-to-pay at period *t*	$\omega (\tau ) \in \mathbb {R}^+$
$\gamma (\tau )$	: Buying time-sensitivity coefficient	$\gamma \in \mathbb {R}^+$
$\lambda (\tau )$	: Product variant sensitivity coefficient, incurred only under MC technology	$\lambda \in \mathbb {R}^+$
$U^{\mathcal {T}}(\xi ,t)$	: Customer $\xi$’s utility at period *t*, dependent on the production strategy $\mathcal {T}$ (1)	
$p_t$	: Selling price at period *t*	$0 \le p_t \le max\{0,U^{\mathcal {T}}\}$, $\forall j \in \Phi$
$*$	: Subscript denoting the demand forecast method	$*=_{c},_{u}$
$_{*}D'_{j,t}$	: Demand forecast of variant *j* at time *t*	
*D* *j*, *t*	: Observed demand for product variant *j* at time *t*	
$I'_{j,t}$	: Target inventory level of variant *j* at time *t*	
*I* *j*, *t*	: Observed inventory level of variant *j* at time *t*	
$K^A$	: Constant production capacity under AM	
$K^M$	: Constant production capacity under MC	Equally distributed among *n*
$\kappa$	: Production capacity magnitude	$\kappa \in \mathbb {R^+}$
$\rho$	: Production capacity ratio between AM and MC	$\rho \in \mathbb {R^+}$
$S_t$	: Sales at period *t*	
$L_t$	: Lost sales at period *t*	
$Q_{j,t}$	: Optimal production quantity for each variant *j* at time *t*	
$c^A$	: Constant marginal production cost under AM	$c^A=$ constant $>0$
$c^b$	: Unit production base cost under MC	$c^b=$ constant $>0$
$c^M(n)$	: Unit production cost under MC depending on *n*	$c^M(n)= c_b(1+(n-1)\delta )>0$
$k^A$	: One-time fixed cost for AM technology	$k^A=$ constant $>0$
$k^M$	: One-time fixed cost for MC technology	$k^M=$ constant $>0$
*h*	: Inventory holding cost per unit per period, common to all product variants	$h \in \mathbb {R^+}$
*s*	: Stockout cost incurred when excess demand is lost per unit of unmet demand,	$h<s$
	common to all product variants	
*v*	: Salvage value of remaining inventory at the end of MC period	$p_t> v > c^M(n)$

## EC.2. Proof of Lemma 1

### Lemma 1

By the Law of Large Numbers (LLN), for all *t*, *j*,

$$\begin{aligned} \left\{ \begin{array}{llll} (i)&{}D_{j,t}/N&{}\rightarrow _{a.s.}&{}{{}_ud'_{j,t}}\\ (ii)&{}D_t/N&{}\rightarrow _{a.s.}&{}{{}_{u}d'_t=\sum _j {}_{u}d'_{j,t}}\\ (iii)&{}I'_{j,t}/N&{}\rightarrow _{a.s.}&{}{i'_{j,t}}\\ (iv)&{}I'_t/N&{}\rightarrow _{a.s.}&{}{i'_t=\sum _j i'_{j,t},\; i'_{j,t}=i'_t/n}\\ (v)&{}I_{j,t}/N&{}\rightarrow _{a.s.}&{}{i_{j,t}}\\ (vi)&{}I_t/N&{}\rightarrow _{a.s.}&{}{i_t=\sum _j i_{j,t}}\\ (vii)&{}Q_{j,t}/N&{}\rightarrow _{a.s.}&{} \left\{ \begin{array}{lll} q_{j,t}&{}=&{}\min ({\tilde{K}}^M/n,\max (i'_t/n-i_{j,t}+_ud'_{j,t}))\text { if }\mathcal {T}(t)=MC,\\ q_{j,t}&{}=&{}0\text { if }{\mathcal {T}(t)=AM,}\\ \end{array} \right. \\ (viii)&{}Q_t/N&{}\rightarrow _{a.s.}&{} \left\{ \begin{array}{lll} q_{t}&{}=&{}\min ({\tilde{K}}^M,\max (i'_t-i_{t}+_ud'_{t}))\text { if }{\mathcal {T}(t)=MC,}\\ q_{t}&{}=&{}0\text { if }{\mathcal {T}(t)=AM,}\\ \end{array} \right. \\ (ix)&{}S_{j,t}/N&{}\rightarrow _{a.s.}&{} s_{j,t}=\min (_ud'_{j,t},q_{j,t}+i_{j,t})\text { if }{\mathcal {T}(t)=MC,}\\ (x)&{}S_t/N&{}\rightarrow _{a.s.}&{} \left\{ \begin{array}{lll} s_{t}&{}=&{}\sum _js_{j,t}=\min ( _ud'_t,i_t+q_t)\text { if }\mathcal {T}(t)=MC,\\ s_t&{}=&{}\min ({\tilde{K}}^A, _u\!d'_t)\text { if }{\mathcal {T}}(t)=AM,\\ \end{array} \right. \\ (xi)&{}L_{j,t}/N&{}\rightarrow _{a.s.}&{}l_{j,t}=\max (0,_ud'_{j,t}-i_{j,t}-q_{j,t})\text { if }{\mathcal {T}(t)=MC;}\\ (xii)&{}L_{t}/N&{}\rightarrow _{a.s.}&{}l_{t}= \left\{ \begin{array}{l} \max (0,_ud'_t-\tilde{K}^A)\text { if }{\mathcal {T}(t)=AM;}\\ \sum _jl_{j,t}=\max (0,_ud'_t-i_t-q_t)\text { if }{\mathcal {T}(t)=MC;}\\ \end{array} \right. \\ (xiii)&{}I_{j,t+1}/N&{}\rightarrow _{a.s.}&{}i_{j,t+1}=\max (0,q_{j,t}+i_{j,t}-s_{j,t})\\ (xiv)&{}I_{t+1}/N&{}\rightarrow _{a.s.}&{}i_{t+1}=\sum _j i_{j,t+1}=\max (0,q_t+i_t-s_t)\\ (xv)&{}\Pi ^\mathcal {T}_t/N&{}\rightarrow _{a.s.}&{} \begin{array}{lll} \pi ^\mathcal {T}_t&{}=&{}s_t(p_t-c_{\mathcal {T}(t)})-sl_t\\ &{}&{}-1_{\mathcal {T}(t)=MC}hi_{t+1}+1_{t=T_{M\rightarrow A}}i_{t+1}(vp_{T_{M\rightarrow A}}-c^M(n))\\ \end{array}\\ \end{array} \right. \end{aligned}$$

Note that $(i'_1,\ldots ,i'_{T+1})$ is also obtained from $(_*d'_1,\ldots ,_*d'_{t})$ by a water filling algorithm (wfs) that resembles the (WFS), described in 4.1:

wfs $$\begin{aligned} \begin{array}{c} \mathtt {(i'_1,\ldots ,i'_{T+1})=wfs}(\mathcal {T},{\tilde{K}}^{\mathtt {M}}\mathtt {,(_{*}d'_1,\ldots ,_{*}d'_T)):}\\ \\ \begin{array}{l} t=T,\;i'_{T+1}=0\\ \mathtt {while }\,\,t>0\\ \qquad \mathtt {if }\,\,t>T_{A\rightarrow M}\\ \qquad \qquad i'_t=\max ((_{*}d'_t-\tilde{K}^M)\varvec{1}_{\{\mathcal {T}(t)=MC\}}+i'_{t+1},0)\\ \qquad \qquad t=t-1\\ \qquad \mathtt {else if \,\,}\le \,\,T_{A\rightarrow M}\\ \qquad \qquad i'_t=0\\ \qquad \qquad t=t-1\\ \qquad \mathtt {end}\\ \mathtt {end}\\ \end{array} \end{array} \end{aligned}$$

### Proof of Lemma 1

By the Law of Large Numbers (LLN), one can obtain the $(i-ii)$ *a.s.* convergence of the mean actual demand to the mean uncensored demand for all *t*, *j*. From this follows the *a.s.* convergence of the quantities involved in the profit function, i.e., the mean of $(iii-iv)$ target, $(v-vi)$ on-hand inventory levels, $(vii-viii)$ production quantity, $(ix-x)$ sales, $(xi-xii)$ lost sales, $(xiii-xiv)$ on-hand inventory for the next period and (*xv*) periodic profits.

Indeed, the convergence in (*ii*) is a direct consequence of the Law of Large Numbers (LLN), since the draw of the random population of size *N* is independent and uniform with law $\mathbb {P}_\xi$, and by (3,4),

$$\begin{aligned}&(D_1,\ldots ,D_T)/N\rightarrow (\varvec{d'}_{\varvec{1}},\ldots ,{\varvec{d'}}_{\varvec{T}})\\&\quad =\left( \mathbb {P}(U^\mathcal {T}(\xi ,t)\ge p_1), \right. \\&\quad \left. \mathbb {P}(\{U^\mathcal {T}(\xi ,t)\ge p_2)\}\cap \{U^\mathcal {T}(\xi ,t)<p_1)\}),\ldots ,\right. \\&\quad \left. \mathbb {P}(\{U^\mathcal {T}(\xi ,t)\ge p_T\}\cap (\cap _{1\le k<T}\{U^\mathcal {T}(\xi ,g)<p_k\}))\right) .\\ \end{aligned}$$

From (*ii*) and (2,6), one deduces (*i*). Next, from the structure of the WFS algorithm (WFS) and the convergences in $(i-ii)$, (*iii*) and (*iv*) follow. Note that at this stage, one can also deduce (wfs).

The remaining statements of convergence follow by induction on *t*: from (17,18) and the convergence of demand, one deduces the convergence of the mean production quantities and inventories, while the limit formulas in (*v*, *vi*, *vii*, *viii*) follow. Further, the convergences of $(ix-xii)$ arises based on the expressions for realized and lost sales of the manufacturer. The convergence and the equation in (*xiii*) follow from (18), while (*xiv*), in turn, follows from (*vi*) and (*xiii*). To conclude, (*xv*) is deduced from the (7,8) and the preceding convergences $(i-xiv)$ for each $1\le r\le t$. $\square$

## EC.3. Proof of Theorem 2

### Proof of Theorem 2

Based on the Lemma 2, one needs to prove that $D_{j,t}/N\rightarrow _ud'_{j,t}$ and $D_t/N\rightarrow _ud'_t$ uniformly in $p\in \mathcal {P}$. The proof of these uniform convergences can be conducted strictly as in ((Lacroix et al., 2021),proof of Theorem 1). The only difference is in considering the mean demand instead of the *mean profit per customer as in *Lacroix et al. (2021). $\square$

## EC.4. Profit algorithm (A-MTS) for Theorem 1

The mean profit per customer per period, $\pi ^\mathcal {T}_t$, is obtained algorithmically as follows

A-MTS $$\begin{aligned} \begin{array}{c} \texttt {(i'}_\texttt {1},\ldots ,\texttt {i'}_{\texttt {T}+\texttt {1}})=\texttt {wfs}(\mathcal {T},{\tilde{K}}^\texttt {M},(_\texttt {*}{} \texttt {d'}_\texttt {1},\ldots ,_\texttt {*}{} \texttt {d'}_\texttt {T}))\\ \begin{array}{l} i_1=0;\\ t=1;\\ \tilde{\pi }(\mathcal {T},n,p)=0\\ \texttt {while}\; t\le T\\ \qquad \texttt {if}\; \mathcal {T}(t)=MC\\ \qquad \qquad q_t=\min (\tilde{K}^M,\max (i'_t-i_t+_ud'_t));\\ \qquad \qquad s_t=\min (_ud'_t,i_t+q_t);\\ \qquad \qquad l_{t}=\max (0,_ud'_t-i_t-q_t);\\ \qquad \qquad i_{t+1}=q_t+i_t-s_t;\\ \qquad \texttt {else}\\ \qquad \qquad q_t=0;\\ \qquad \qquad s_t=\min (\tilde{K}^A, _u\! d'_t);\\ \qquad \qquad l_t=\max (0,_ud'_t-\tilde{K}^A);\\ \qquad \qquad i_{t+1}=i_t;\\ \qquad \texttt {end}\\ \qquad \pi ^\mathcal {T}_t=s_t(p_t-c_{\mathcal {T}(t)})-sl_t-\varvec{1}_{\{\mathcal {T}(t)=MC\}} h i_{t+1}\\ \qquad \qquad \qquad +\varvec{1}_{\{t=T_{M\rightarrow A}\}}i_{t+1}(vp_{T_{M\rightarrow A}}-c_{\mathcal {T}(t)})\\ \qquad \tilde{\pi }(\mathcal {T},n,p)=\tilde{\pi }(\mathcal {T},n,p)+ \pi ^\mathcal {T}_t;\\ \qquad t=t+1;\\ \texttt {end}\\ \tilde{\pi }(\mathcal {T},n,p)=\tilde{\pi }(\mathcal {T},n,p)-\tilde{k}^M\varvec{1}_{\{T_{A\rightarrow M}<T\}}\\ \qquad \qquad -\tilde{k}^A \varvec{1}_{\{T_{A\rightarrow M}>0\text { or }T_{M\rightarrow A}<T\}}\\ \end{array} \end{array} \end{aligned}$$

## EC.5. Profit algorithm (A-MTOC) for corollary 1

The limit of the *mean profit per customer* is obtained by a simplified version of the (A-MTS) algorithm (see EC.4):

A-MTOC $$\begin{aligned}&t=1;\\&\tilde{\pi }(\mathcal {T},n,p)=0;\\&\texttt {while}\; t\le T\\&\qquad s_t=\min (_ud'_t,\tilde{K}^{\mathcal {T}(t)});\\&\qquad l_t=\max (0,_ud'_t-\tilde{K}^{\mathcal {T}(t)});\\&\qquad \pi ^\mathcal {T}_t=s_t(p_t-c_{\mathcal {T}(t)})-sl_t;\\&\qquad \tilde{\pi }(\mathcal {T},n,p)=\tilde{\pi }(\mathcal {T},n,p)+ \pi ^\mathcal {T}_t;\\&\qquad t=t+1;\\&\texttt {end}\\&\tilde{\pi }(\mathcal {T},n,p)=\tilde{\pi }(\mathcal {T},n,p)-\tilde{k}^M\varvec{1}_{\{T_{A\rightarrow M}<T\}}\\&\qquad -\tilde{k}^A \varvec{1}_{\{T_{A\rightarrow M}>0\text { or }T_{M\rightarrow A}<T\}} \\ \end{aligned}$$

## EC.6. Profit algorithm (A-MTOUC) for Corollary 2

Similarly, the limit of the *mean profit per customer* is obtained by a simplified version of the (A-MTS) algorithm (see EC.4):

A-MTOUC $$\begin{aligned}&t=1;\\&\tilde{\pi }(\mathcal {T},n,p)=0;\\&\texttt {while}\; t\le T\\&\qquad s_t=_ud'_t;\\&\qquad \pi ^\mathcal {T}_t=s_t(p_t-c_{\mathcal {T}(t)})\\&\qquad \tilde{\pi }(\mathcal {T},n,p)=\tilde{\pi }(\mathcal {T},n,p)+ \pi ^\mathcal {T}_t;\\&\qquad t=t+1;\\&\texttt {end}\\&\tilde{\pi }(\mathcal {T},n,p)=\tilde{\pi }(\mathcal {T},n,p)-\tilde{k}^M\varvec{1}_{\{T_{A\rightarrow M}<T\}}\\&\quad -\tilde{k}^A \varvec{1}_{\{T_{A\rightarrow M}>0\text { or }T_{M\rightarrow A}<T\}} \\ \end{aligned}$$

## References

[CR1] AFMG. (2020). The benefits of 3d printing for consumer goods. https://amfg.ai/industrial-applications-of-3d-printing-the-ultimate-guide/.

[CR2] Alptekinoğlu A, Corbett CJ (2008). Mass customization vs. mass production: Variety and price competition. Manufacturing & Service Operations Management.

[CR3] Anderson, D. M. (2004). *Build-to-Order & Mass Customization: The ultimate supply chain management and lean manufacturing strategy for low-cost on-demand production without forecasts or inventory*. CIM Press.

[CR4] Arbabian ME, Wagner MR (2020). The impact of 3D printing on manufacturer-retailer supply chains. European Journal of Operational Research.

[CR5] Bass FM (1969). A new product growth for model consumer durables. Management Science.

[CR6] Bass FM (2004). Comments on a “new product growth for model consumer durables the bass model”. Management Science.

[CR7] Baumers M, Dickens P, Tuck C, Hague R (2016). The cost of additive manufacturing: Machine productivity, economies of scale and technology-push. Technological Forecasting and Social Change.

[CR8] Berman Barry (2012). 3-d printing: The new industrial revolution. Business Horizons.

[CR9] Campbell, I, O Diegel, R Huff, J Kowen. (2020). Wohlers report 2020: 3d printing and additive manufacturing state of the industry. *Wohlers Associates* .

[CR10] Chatterjee Rabik Ar, Eliashberg Jehoshua (1990). The innovation diffusion process in a heterogeneous population: A micromodeling approach. Management Science.

[CR11] Chen, Li., Cui, Yao, & Lee, Hau L. (2020). *On-Demand Customization and Channel Strategies* (pp. 165–192). Springer International Publishing.

[CR12] Chinneck, J W. (2015). *Practical optimization: A gentle introduction. Chapter 17: Pattern Search for Unconstrained NLP*. Systems and Computer Engineering, Carleton University, Ottawa. http://www.sce.carleton.ca/faculty/chinneck/po/Chapter17.pdf.

[CR13] Davies, S. (2018). Bowman international manufactures bespoke rollertrain bearing cages with multi jet fusion https://www.tctmagazine.com/additive-manufacturing-3d-printing-news/bowman-rollertrain-bearing-cages-multi-jet-fusion/.

[CR14] Deradjat D, Minshall T (2017). Implementation of rapid manufacturing for mass customisation. Journal of Manufacturing Technology Management.

[CR15] Dong, L., Shi, D., Zhang, F. (2020). 3d printing and product assortment strategy. Working paper, Washington University in St. Louis, St. Louis. Available at SSRN 2847731.

[CR16] Foulkes, N. (2022). Omega and swatch surprise the market with a 207 moonswatch. *Financial Times, *https://www.ft.com/content/7ca0dac6-48ea-4ccf-af36-468a9cac3558 .

[CR17] Graves Stephen C (1999). A single-item inventory model for a nonstationary demand process. Manufacturing & Service Operations Management.

[CR18] Hadley G, Whitin TM (1961). An optimal final inventory model. Management Science.

[CR19] Ho T-H, Savin S, Terwiesch C (2002). Managing demand and sales dynamics in new product diffusion under supply constraint. Management Science.

[CR20] Holweg, M. (2015). The limits of 3d printing. *Harvard Business Review: Digital Review Articles* 2–4.

[CR21] Hon, K.K.B. (2007). Digital additive manufacturing: from rapid prototyping to rapid manufacturing. In *Proceedings of the 35th International MATADOR Conference*. Springer, pp 337–340.

[CR22] Ideo. (2020). What is design thinking? https://www.ideou.com/blogs/inspiration/what-is-design-thinking.

[CR23] Jiang K, Lee HL, Seifert RW (2006). Satisfying customer preferences via mass customization and mass production. IIE Transactions.

[CR24] Kök, A., Fisher, M. L., & Ramnath, V. (2015). Assortment planning: Review of literature and industry practice. In A. Narendra & S. A. Smith (Eds.), *Retail supply chain management: Quantitative models and empirical studies* (pp. 175–236). Boston. 10.1007/978-1-4899-7562-1_8

[CR25] Kumar S, Swaminathan JM (2003). Diffusion of innovations under supply constraints. Operations Research.

[CR26] Kurawarwala AA, Matsuo H (1996). Forecasting and inventory management of short life-cycle products. Operations Research.

[CR27] Lacroix R, Seifert RW, Timonina-Farkas A (2021). Benefiting from additive manufacturing for mass customization across the product life cycle. Operations Research Perspectives.

[CR28] Lancaster K (1990). The economics of product variety: A survey. Marketing Science.

[CR29] MX3D. (2020). Print on demand https://mx3d.com/print-on-demand/.

[CR30] Rogers H, Baricz N, Pawar KS (2016). 3D printing services: Classification, supply chain implications and research agenda. International Journal of Physical Distribution & Logistics Management.

[CR31] Sasson A, Johnson JC (2016). The 3D printing order: Variability, supercenters and supply chain reconfigurations. International Journal of Physical Distribution & Logistics Management.

[CR32] Sethuraman, N., Parlakturk, A. K., Swaminathan, J. M. (2018). Personal fabrication as an operational strategy: Value of delegating production to customer. *Kenan Institute of Private Enterprise Research Paper No. 18-5* .

[CR33] Shapiro, Alexander, Dentcheva, Darinka, & Ruszczynski, Andrzej. (2014). *Lectures on stochastic programming: Modeling and theory* (2nd ed.). Society for Industrial and Applied Mathematics.

[CR34] Shen W, Duenyas I, Kapuscinski R (2013). Optimal pricing, production, and inventory for new product diffusion under supply constraints. Manufacturing & Service Operations Management.

[CR35] Snyder, L. V., & Shen, Z. J. M. (2019). *Fundamentals of supply chain theory*. Wiley.

[CR36] Song J-S, Zhang Y (2020). Stock or print? Impact of 3-d printing on spare parts logistics. Management Science.

[CR37] Timonina-Farkas A, Katsifou A, Seifert R (2020). Product assortment and space allocation strategies to attract loyal and non-loyal customers. European Journal of Operational Research.

[CR38] Treharne JT, Sox CR (2002). Adaptive inventory control for nonstationary demand and partial information. Management Science.

[CR39] Weller C, Kleer R, Piller FT (2015). Economic implications of 3d printing: Market structure models in light of additive manufacturing revisited. International Journal of Production Economics.

[CR40] Westerweel, B., Basten, R., van Houtum, G. J. (2018a). Printing spare parts at remote locations: Fulfilling the promise of additive manufacturing. Working paper, Eindhoven University of Technology, Eindhoven.

[CR41] Westerweel B, Basten RJI, van Houtum G-J (2018). Traditional or additive manufacturing? Assessing component design options through lifecycle cost analysis. European Journal of Operational Research.

[CR42] Yang YH, Kim JS (2018). An adaptive joint replenishment policy for items with non-stationary demands. Operational Research an International Journal.

[CR43] Yu, W., Cioffi, J. M. (2001). On constant power water-filling. In *ICC 2001. IEEE International Conference on Communications. Conference Record (Cat. No. 01CH37240)*, vol. 6. IEEE, pp 1665–1669.

